# Exercise for reducing chemotherapy-induced peripheral neuropathy: a systematic review and meta-analysis of randomized controlled trials

**DOI:** 10.3389/fneur.2023.1252259

**Published:** 2024-01-12

**Authors:** Yingjie Huang, Tian Tan, Lu Liu, Zijian Yan, Yuexia Deng, Guangyao Li, Min Li, Jia Xiong

**Affiliations:** ^1^Guangzhou University of Chinese Medicine, Guangzhou, China; ^2^Clinical Medical College of Acupuncture Moxibustion and Rehabilitation, Guangzhou University of Chinese Medicine, Guangzhou, China; ^3^The First Clinical College of Guangzhou University of Chinese Medicine, Guangzhou, China; ^4^Southern Theater General Hospital, Guangzhou, China; ^5^Department of Traditional Chinese Medicine, Guangdong Provincial Key Laboratory ofMajor Obstetric Diseases, Guangdong Provincial Clinical Research Center for Obstetrics and Gynecology, The Third Affiliated Hospital of Guangzhou Medical University, Guangzhou, China; ^6^Nanchang Hongdu Hospital of Traditional Chinese Medicine, Nanchang, Jiangxi Province, China

**Keywords:** exercise therapy, chemotherapy-induced peripheral neuropathy, CIPN, efficacy, meta-analysis

## Abstract

**Background:**

More than half of cancer patients develop severe chemotherapy-induced peripheral neuropathy (CIPN), resulting in low quality of life, negative effects on function, and challenges in treatment compliance. Most recent studies have shown that exercise therapy has a positive impact on reducing CIPN symptoms and can also improve quality of life, balance, and activity levels. The aim of this meta-analysis was to evaluate the effect of exercise therapy on the efficacy of CIPN.

**Methods:**

Computerized search of Embase, Web of Science, CNKI, Wan Fang Data, VIP, CBM for RCTs on exercise therapy for CIPN from database creation to November 2022, without language restriction. The Cochrane Handbook 5.3 risk of bias assessment tool was used to evaluate the quality of the included studies. Then Revman 5.3 software was used to evaluate the quality of the included studies. The heterogeneity of the research results is tested by I^2^, continuous variables were presented as weighted mean difference or standard mean difference, and confidence intervals were set at 95%. Stata15.0 was utilized to conduct a meta-analysis.

**Results:**

A total of 15 RCTs with 1,124 patients were included. Meta-analysis showed that the test group was superior to the control group in terms of total symptom score (SMD: −0.62; 95% Cl: −0.99, −0.24), numbness, tingling, quality of life score (total score, physical, function), pain, balance, and neurotoxicity function assessment (FACT/GOG-NTX) questionnaire (*p* < 0.05).

**Limitations:**

The duration and frequency of treatment are different every week, which may have some impact on the results.

**Conclusion:**

Exercise therapy can be effective in treating CIPN by improving symptom score (total symptom score, numbness, tingling), quality of life score (total score, physical function), pain, balance, and FACT/GOG-NTX questionnaires. It still needs to be refined and validated by more high-quality, multicenter, large-sample RCTs in the future.

**Systematic review registration:**

https://www.crd.york.ac.uk/prospero/display_record.php?ID=CRD42022373131, identifier: CRD42022373131.

## 1 Introduction

Cancer is one of the leading causes of death worldwide (1, 2). According to relevant research statistics, by 2040, the annual number of new cancer cases is expected to rise to 29.5 million, and the number of cancer-related deaths will increase to 16.4 million [Global Cancer Observatory (Fig. iarc.fr)]. Chemotherapy is the most common and effective method for treating cancer. However, more than half all-cause cancer patients have developed severe peripheral neuropathies (3) during chemotherapy treatment which called chemotherapy-induced peripheral neuropathy (CIPN). CIPN is caused by multiple chemotherapeutic drugs that can damage sensory, motor, autonomic, or cranial nerves and typically manifest as numbness, loss of proprioception sense, tingling, burning painpins, and hyperalgesia present (4). The insufficient awareness of its pathogenesis, appropriate dosage and benefits of rehabilitation in reducing the severity of CIPN, leads to low patients’ quality of life, negative impacts on functional outcomes and challenges of treatment compliance (5).

The neurotoxic alterations in intracellular organelles, membrane receptors and ion channels, induced by chemotherapeutic agents will cause alterations of the intracellular homeostasis, signaling and neurotransmission, finally leading to the occurrence of CIPN (6). Unfortunately, we have not found treatments for CIPN, and the current first-line drugs used to treat CIPN shows mild or unsatisfactory therapeutic efficacy. Symptomatic treatment gabapentin, nortriptyline and amitriptyline which use to treat neuropathic pain show only modest or no benefits in some randomized, double-blinded, placebo controlled crossover study on CIPN (7–9). Only duloxetine suggested definite effect to relief neuropathic pain in CIPN in recent studies (10). Cumulated evidence indicates that ion channels plays a critical role in the pathology of painful CIPN, therefore ion channel blocker may be a promising candidate for concomitant use against CIPN, but there still remains few convincing evidence supporting its efficacy (11).

According to ASCO guideline (2), in the context of a clinical trial, non-pharmacological options for patients who have completed neurotoxic chemotherapy include exercise, acupuncture, and scrambler therapy, but larger sample-sized conclusive studies are needed to confirm efficacy and clarify risks. Given low risk of harm, these non-pharmacological options also received weak recommendations in The European Society of Medical Oncology guidelines (12).

Exercise therapy is a simple and cost-effective rehabilitation method. Exercise therapy is an intervention to improve CIPN symptoms through proper stretching followed by aerobic training and/or strength training of major muscle groups. Its types broadly include strength training, aerobic exercise, stretching, sensory training (13). Most recent studies have shown that exercise therapy has a positive impact on decreasing peripheral neuropathy symptoms and also can improve quality of life, balance, and activity levels (14). Analyzing its mechanism, exercise can up regulate the levels of multiple protective Neurotrophin, including glial Neurotrophin, Brain-derived neurotrophic factor and Insulin-like growth factor, in order to promote the growth, survival and differentiation of mature and developing neurons, change the vascular function and metabolic activity of peripheral nerves, reduce inflammation and alleviate CIPN symptoms (15). Currently, multicenter, large-sample randomized controlled trials remain lacking, and there is no sufficient evidence-based evidence of exercise on CIPN symptoms in cancer patients. Therefore, this study demonstrated through systematic review and meta-analysis that exercise therapy treats CIPN by improving symptom scores, quality of life scores, pain, balance, FACT/GOG-NTX questionnaire, and provide evidence-based medical conclusion to improve this clinical problem.

## 2 Methods

We have registered this study with the PROSPERO international prospective register of systematic reviews (CRD42022373131). This study was conducted as claimed by the recommendations of the Cochrane and follows the Preferred Reporting Items for Systematic Reviews and Meta-Analysis (PRISMA) guidelines (16).

### 2.1 Inclusion criteria

The population-intervention Comparators-Outcomes-Study design (PICOS) framework was used as the eligibility criteria for the review as follows.

#### 2.1.1 Selection of studies

All randomized controlled trials (RCT) investigating exercise therapy for CIPN without language restriction.

#### 2.1.2 Selection of participants

The study subjects were patients clinically diagnosed with peripheral neuropathy caused by cancer chemotherapy, and all chemotherapy patients had symptoms of peripheral neuropathy, which did not limit the types of cancer and chemotherapeutic drugs.

#### 2.1.3 Types of interventions

All of the patients received chemotherapy and had CIPN. The experimental group was treated with exercise therapy, including stretching exercise, strength training, yoga, balance exercise, sensory exercise, aerobic exercise. No restriction on the type of exercise therapy, one or more exercises can be used. The control group was treated with usual care or health education. Studies that did not meet the above inclusion criteria were excluded. In addition, the following exclusion criteria were used: ① not randomized controlled trial; ② peripheral neuropathy caused by other diseases in patients; ③ patients had recovered from CIPN after the end of chemotherapy; ④ CIPN patients received duloxetine; ⑤ studies without original data or incomplete study data; ⑥ studies with interventions that did not meet the inclusion criteria.

### 2.2 Outcome measures

Through the review of clinical trials published in major databases and academic journals to evaluate CIPN, We found that the primary outcome was symptom score (total symptom score, numbness, tingling), the secondary outcome includes quality of life score (total score, physical, functional, social, emotional, neurotoxicity), pain, balance, and functional assessment of neurotoxicity (FACT/GOG-NTX) questionnaire.

### 2.3 Database search

PubMed, Embase, Web of Science, China National Knowledge Infrastructure (CNKI), Wan Fang Data Knowledge Service Platform (Wan Fang Data), China Science and Technology Journal Database (VIP),Chinese biomedical literature database(CBM) were searched by computer from database establishment to November 2022.The search strategy used the following keywords: “chemotherapy-induced peripheral neurotoxicity,” “CIPN,” “exercise therapy,” “Exercise,” and “Rehabilitation.” Taking PubMed as an example, search terms and strategies were as follows: [(chemotherapy-induced neurotoxicity) OR (chemotherapy-induced peripheral neuropathy) OR (CIPN)] AND [(exercise therapy) OR (Exercise Therapies)] OR (Therapies, Exercise) OR (Rehabilitation Exercise) OR (Exercise, Rehabilitation) OR (Rehabilitation Exercise).

### 2.4 Literature screening and data extraction

Two investigators (YH, TT) independently performed literature screening in strict accordance with the inclusion and exclusion criteria, and the retrieved articles were managed and identified using Note Express software. Excel software was used to establish literature information extraction data, including study type, number of cases, age, cancer type, chemotherapeutic drugs used, disease duration, intervention treatment duration, and outcome measures. Finally, findings were cross-checked and disputes resolved by discussion or consultation with third parties.

In cases where primary data is unavailable or presented graphically in studies, attempts will be made to contact the respective authors to request the primary data, and the data in the figures will be extracted using GetData Graph Digitizer 2.26.

### 2.5 Quality assessment

The Cochrane Handbook 5.3 risk of bias assessment tool was used to evaluate the quality of the included studies, while Revman 5.3 was used to evaluate the methodological quality of the articles. These include: (1) random sequence generation; (2) allocation concealment; (3) blinding of subjects and study personnel; (4) blinding of results evaluators; (5) completeness of outcome data; (6) selective reporting of study results; and (7) other biases. The evaluation results were expressed as “low risk of bias,” “unclear risk of bias” and “high risk of bias.”

### 2.6 Certainty assessment

GDT software (17) was used to assess the certainty of evidence according to the GRADE guidelines (gradeworkinggroup.org) for primary outcomes based on areas of study design, risk of bias, inconsistency, indirectness, imprecision, and other considerations, such as publication bias, effect size, and potential confounding. And the quality of the final evidence was classified as high, moderate, low and very low (18, 19).

### 2.7 Statistical analysis

Stata 15.0 software was used to perform the meta-analysis. Continuous variables were analyzed by the inverse variance approach, with the weighted mean difference (WMD) used as the effect indicator. When the absolute difference of continuous variables is large or the unit is uneven, the standardized mean difference (SMD) was used as the effect indicator. And the confidence intervals (CI) set at 95%. Heterogeneity of study results was tested using I^2^. Results data from the fixed effects model (FE) were selected for analysis if I^2^ ≤ 50%; results data from the random effects model (RE) were selected for combined analysis if I^2^ > 50%. Subgroup meta-analysis and sensitivity analysis was used to analyze the source of heterogeneity and assess the stability of meta-analysis results. Funnel plots were used to analyze the publication bias of the studies.

## 3 Results

### 3.1 Search results

**Figure 1:**
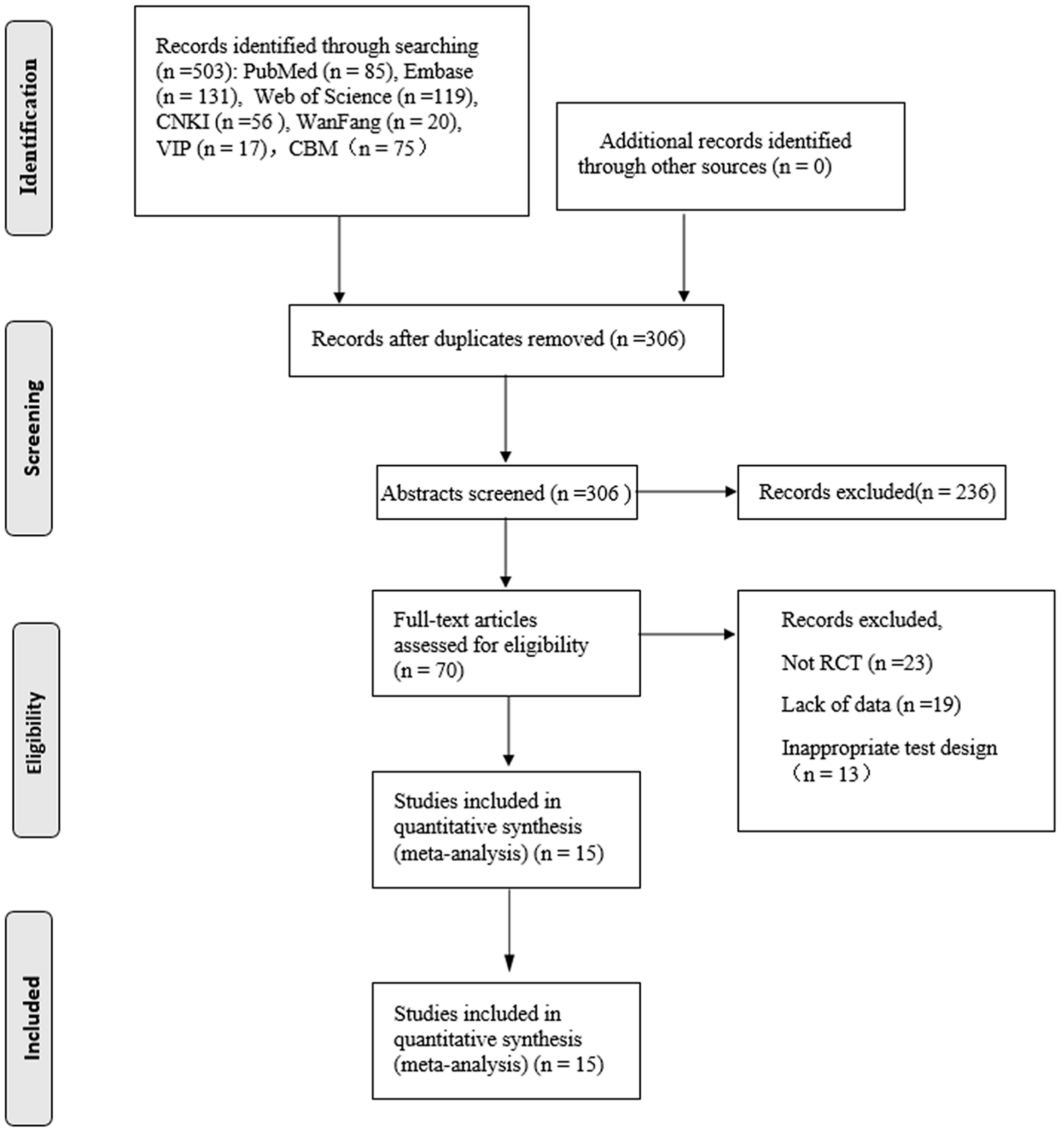
The inclusion process of literature.

According to the search strategy, 503 studies were retrieved. After exclusion of 197 duplicate studies, abstracts and titles of 306 studies were carefully read. After exclusion of titles and abstracts, 70 studies were read in full. After full-text evaluation, 55 studies were excluded for the following reasons: not RCTs (*n* = 23), lack of data (*n* = 19), and Inappropriate test design (*n* = 13). Eventually,15 studies (14, 20–33)and 1,124 patients were included in this Meta-analysis. The studies included in this meta-analysis were multicenter, mainly from the United States, Germany, and China. 13 studies in English and 2 studies in Chinese. Exercise therapy includes stretching, balance, yoga, sensory, strength training, aerobic exercise. The duration of each exercise is 15-60 min, mainly focusing on 30 min, 60 min. The frequency of exercise is mainly 1 time/day, 2 times/week, 3 times/week, 5–7 times /week. The intervention time was 2–36 weeks, mainly 6 weeks and 12 weeks. The patient’s cancer types were Breast/Lymphoma/Colon/Lung/Hematological malignancy/ Gastrointestinal / Ovarian / Cervical cancer, and the chemotherapeutic drugs used were mainly Taxane and Platinum (Table 1). The PRISMA flowchart shows this process (Figure 1).

**Table 1:** The basic characteristics of the included studies.

Trail	Country	Sample size (T/C)	Age(y), Mean ± SD or Median (Range)		Duration/W		T	C	Main outcomes	Frequency	Follow-up time/week	Cancer	Chemotherapy
			T	C	T	C							
Şimşek et al. (29)	Turkey	30,30/30	>18y	>8y	NA	NA	Stretching exercise+balance exercise	Chemotherapy+usual care	①②	5 times/Week	12	Breast cancer	Taxane
Kleckner et al. (25)	USA	170/185	55.6 ± 11.8	55.9 ± 9.7	12.1 ± 60.9	5.7 ± 10.0	Strength training	Chemotherapy	①③	60 min, qd	6	Breast/Lymphoma/Colon/Lung cancer	Taxane-, platinum-, or vinca alkaloid
Ikio et al. (24)	Japan	19/20	69(60–89)	64(57–87)	5(2–111)	6(2–93)	Strength training	Chemotherapy	①②	30 min, tiw	NA	Hematological malignancy/Gastrointestinal cancer	Vinca alkaloids, taxanes, platinum compounds and proteasome inhibitors
Gui et al. (23)	China	51/28	50 ± 8	52 ± 7	12(23.5)	6(21.4)	Sensory exercise	Chemotherapy	②③⑤	qd	2	Digestive malignancies	Oxaliplatin
Bao et al. (21)	USA	21/20	60.0(35.5, 77.9)	62.3(42.4, 79.0)	3.1 (0.5, 10.4)	3.7 (0.9, 15.3)	Yoga	Chemotherapy+usual care	①③⑤	60 min, qd	8	Breast/Uterine/Ovarian cancer	Taxane-, platinum-, or vinca alkaloid
Dhawan et al. (22)	India	22/23	50.5 ± 7.9	52.5 ± 6.6	10.2 ± 7.8	9.8 ± 8.6	Strength training	Chemotherapy	①②③	30 min, qd	10	Ovarian/Cervical/Lung cancer	Paclitaxel, carboplatin
Müller et al. (27)	Germany	49, 57/57	51.7 ± 10.8/53.4 ± 11.7	54.5 ± 11.9	22.0 ± 9.3/23.5 ± 8.9	23.0 ± 9.4	Sensory exercise/Strength training	Chemotherapy	①	35 min, tiw	NA	NA	Taxanes, platinum derivatives, vinca alkaloids
Schwenk et al. (28)	USA	11/11	68.73 ± 8.72	71.82 ± 8.85	49.91 ± 44.11	44.63 ± 56.78	Balance exercise	Chemotherapy+health education	④	45 min, biw	4	NA	NA
Vollmers et al. (31)	Germany	17/19	48.56 ± 11.94	52.39 ± 10.14	12	12	Sensory exercise	Chemotherapy+health education	④	60 min, biw	12	Breast cancer	Taxane
Zimmer et al. (33)	Germany	17/13	68.53(50–81)	70.00(50–81)	27.94 ± 24.557	24.38 ± 19.692	Strength training+balance exercise	Chemotherapy+health education	②④⑤	60 min, biw	8	Colon cancer	Bevacizumab, regorafenib, trastuzumab
Huang et al. (26)	China	32/34	54.56 ± 9.4	57.65 ± 9.8	4.66 ± 1.10	4.79 ± 1.06	Aerobic exercise	Chemotherapy+usual care	①②③	30 min, biw	6	Ovarian cancer	Taxane-, platinum-, or vinca alkaloid
Peng et al. (32)	China	23/23	53.17 ± 9.29	52.15 ± 7.25	NA	NA	Balance exercise	Chemotherapy+usual care	①②③⑤	15-30 min, tiw	12	Breast/Lymphoma cancer/Myeloma	Taxanes, platinum, vincristine, bortezomib, thalidomide
Visovsky et al. (30)	USA	19/19	48.8(24–65)	48.8(24–65)	NA	NA	Aerobic exercise+strength training	Chemotherapy+health education	①②④	30 min/time, 5–7 times/Week	12	Breast cancer	Taxane
Streckmann et al. (34)	Germany	28/28	44(20–67)	48(19–73)	NA	NA	Aerobic exercise+strength training	Chemotherapy	②③	biw	36	Lymphoma cancer	Taxanes,platinum, vincristine, bortezomib, thalidomide
Andersen et al. (20)	Canada	22/26	56.3 ± 9.9	53 ± 10.3	NA	NA	Stretching exercise	Chemotherapy+usual care	①	Four times a week	6	Breast cancer	Taxane

T, trial group; C, control group; NA, not reported; ① symptom score (total symptom score, numbness, and tingling); ② quality of life score (total score, physical, functional, social, emotional, and neurotoxicity); ③ pain; ④ balance; ⑤ functional assessment of neurotoxicity (FACT/GOG-NTX); qd, once a day; biw, 2 times/week; tiw, 3 times/week.

### 3.2 Risk of bias assessment

**Figure 2:**
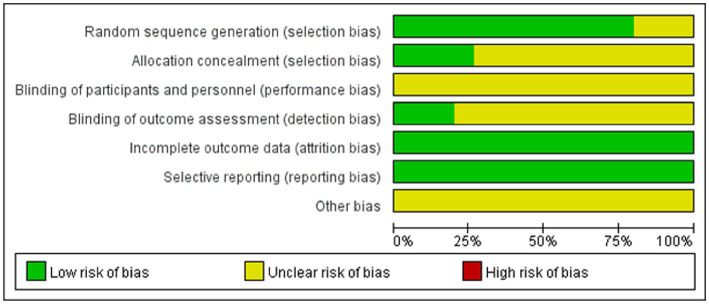
Risk of bias assessment in studies.

The quality of the included studies was moderate. Thirteen studies involved two-arm design (14, 20–26, 28, 30–33),two studies involved three-arm design (28, 29). Twelve studies reported the correct method of generation (random number table or opaque sealed envelope drawing) with a low risk of bias (14, 20–22, 24, 26–30, 32, 33). Three trials did not clearly describe the method of randomization (23, 25, 31). Because of the particularities of the interventions, none study mentioned blinding of patients. Four trials (20, 25, 28, 32) reported allocation concealment. Blinding of outcome assessment was reported in four trials. None of the studies outcomes were incomplete and the risk of bias was low. None of the studies were selectively reported, and other bias were unclear (Figure 2; Supplementary Figure 1).

### 3.3 Main outcome

#### 3.3.1 Symptom score

**Figure 3:**
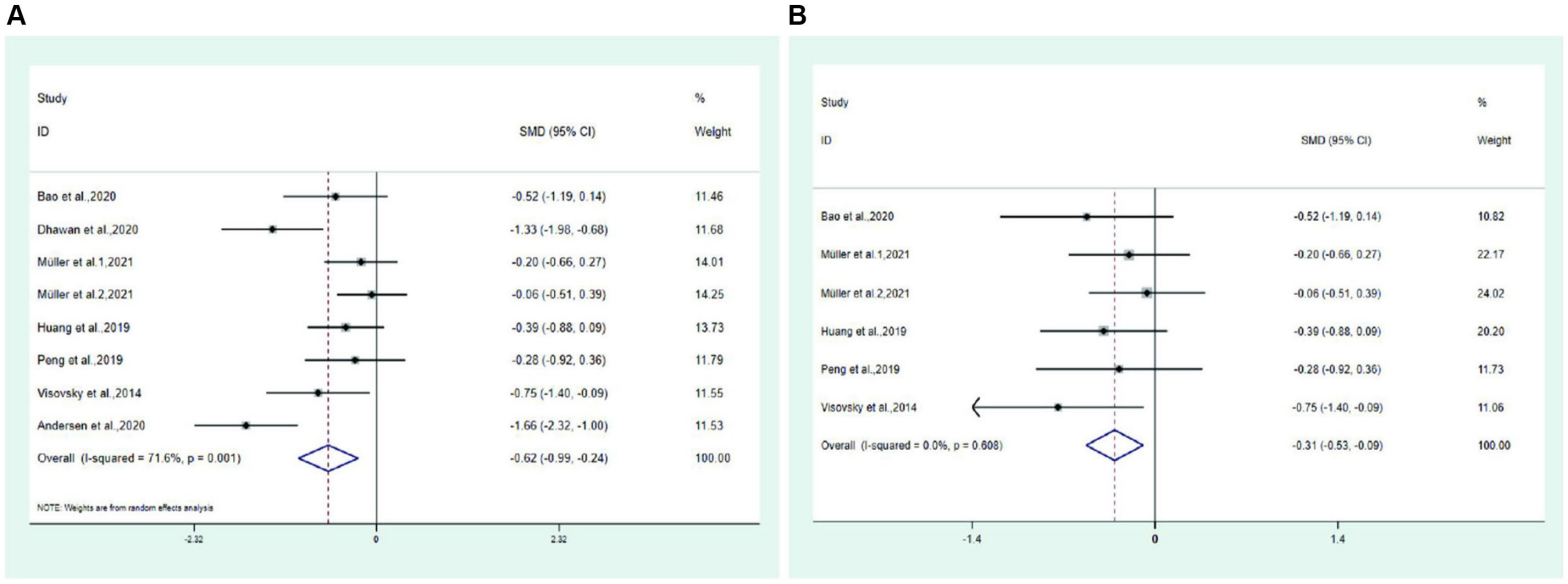
Forest plot. **(A)** Total symptom score. **(B)** Total symptom score after excluding two trail.

① Total symptom score Seven studies (20–22, 26, 27, 30, 32) reported total symptom scores in the test and control groups. Meta-analysis showed that the total symptom score of the test group was significantly lower than that of the control group (SMD: −0.62; 95% Cl: −0.99, −0.24; I^2^ = 71.6%, Figure 3A). The results showed high heterogeneity, so we performed sensitivity analysis (Supplementary Figure 2) and subgroup meta-analysis to analyze the source of heterogeneity. Sensitivity analysis showed that the included trials (20, 22) had a more significant impact on the results. The remaining studies made a meta-analysis, and the new result showed that the total symptom score of the test group was significantly lower than that of the control group (SMD: −0.31; 95% Cl: −0.53, −0.09; I^2^ = 0.0%, Figure 3B).

**Figure 4:**
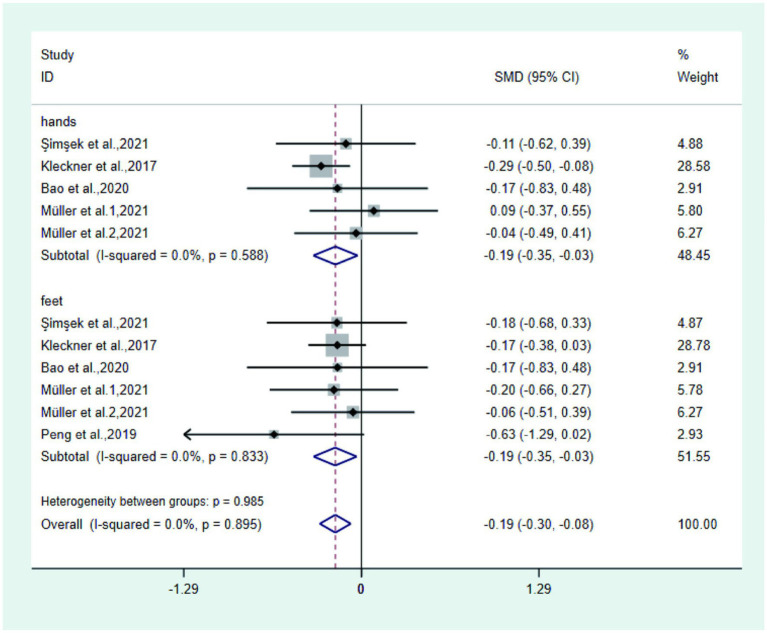
Forest plot of numbness.

② Numbness Five studies (21, 25, 27, 29, 32) reported numbness in the test and control groups, four studies (21, 25, 27, 29) reported numbness in the hands and feet, and one study (32) only reported numbness in the feet. Meta-analysis showed that the numbness of hands and feet in the test group was significantly lower than that in the control group (SMD: − 0.19; 95% Cl: − 0.30, − 0.08; I^2^ = 0.0%, Figure 4). Among them, hand numbness was significantly lower in the test group than in the control group (SMD: − 0.19; 95% Cl: − 0.35, − 0.03; I^2^ = 0.0%), and foot numbness was significantly lower in the test group than in the control group (SMD: − 0.19; 95% Cl: − 0.35, − 0.03; I^2^ = 0.0%).

**Figure 5:**
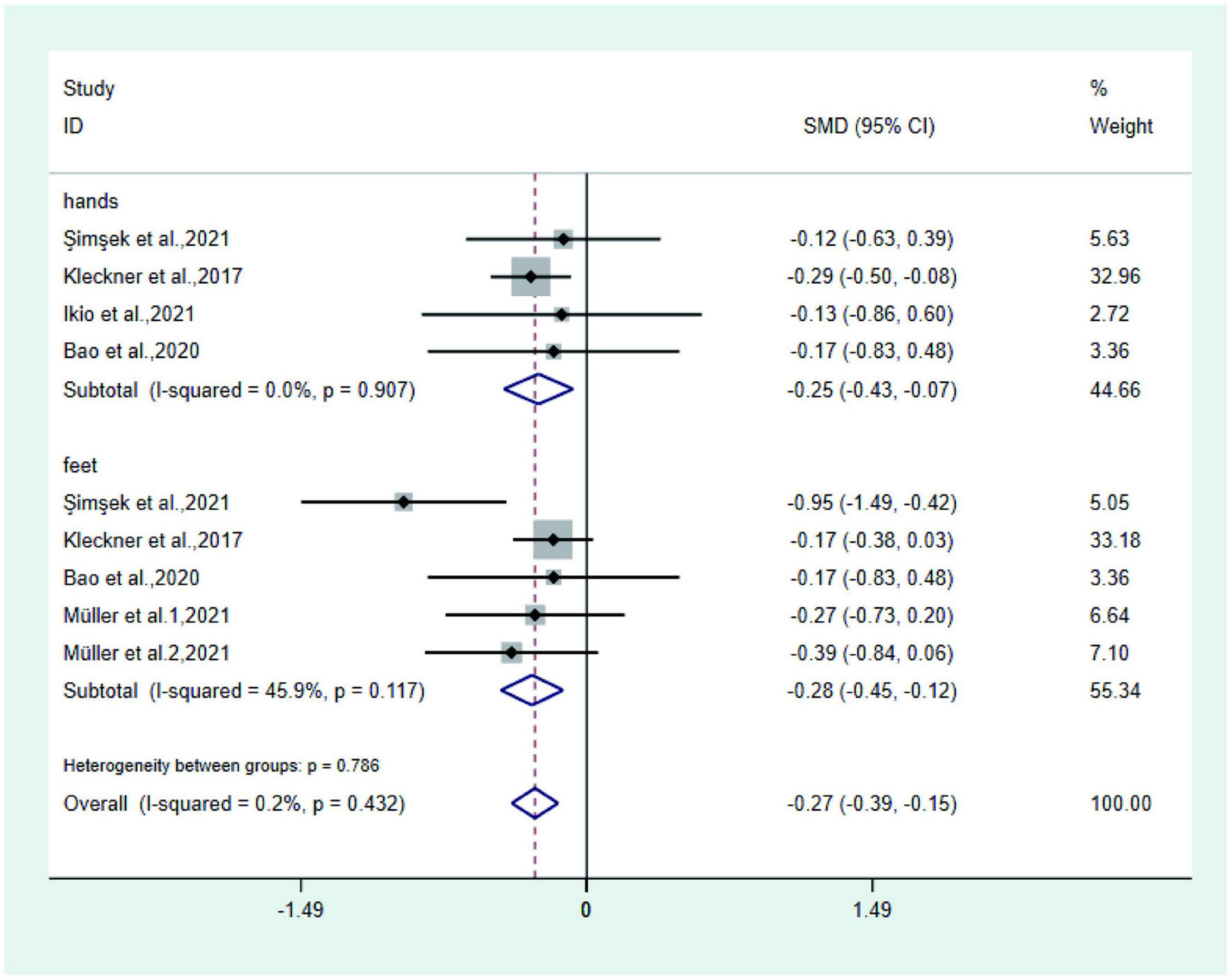
Forest plot of tingling.

③ Tingling Five studies (21, 24, 25, 27, 29) reported tingling in the test and control groups, three studies (21, 25, 29) reported tingling in the hands and feet, one study (24) only reported tingling in the hands, and one study (11) only reported tingling in the feet. Meta-analysis showed that the tingling of hands and feet in the test group was significantly lower than that in the control group (SMD: − 0.27; 95% Cl: − 0.39, − 0.15; I^2^ = 0.2%, Figure 5). Among them, the hand tingling in the test group was significantly lower than that in the control group (SMD: − 0.25; 95% Cl: − 0.43, − 0.07; I^2^ = 0.0%), and the foot tingling sensation in the test group was significantly lower than that in the control group (SMD: − 0.28; 95% Cl: − 0.45, − 0.12; I^2^ = 45.9%).

#### 3.3.2 Quality of life score

**Figure 6:**
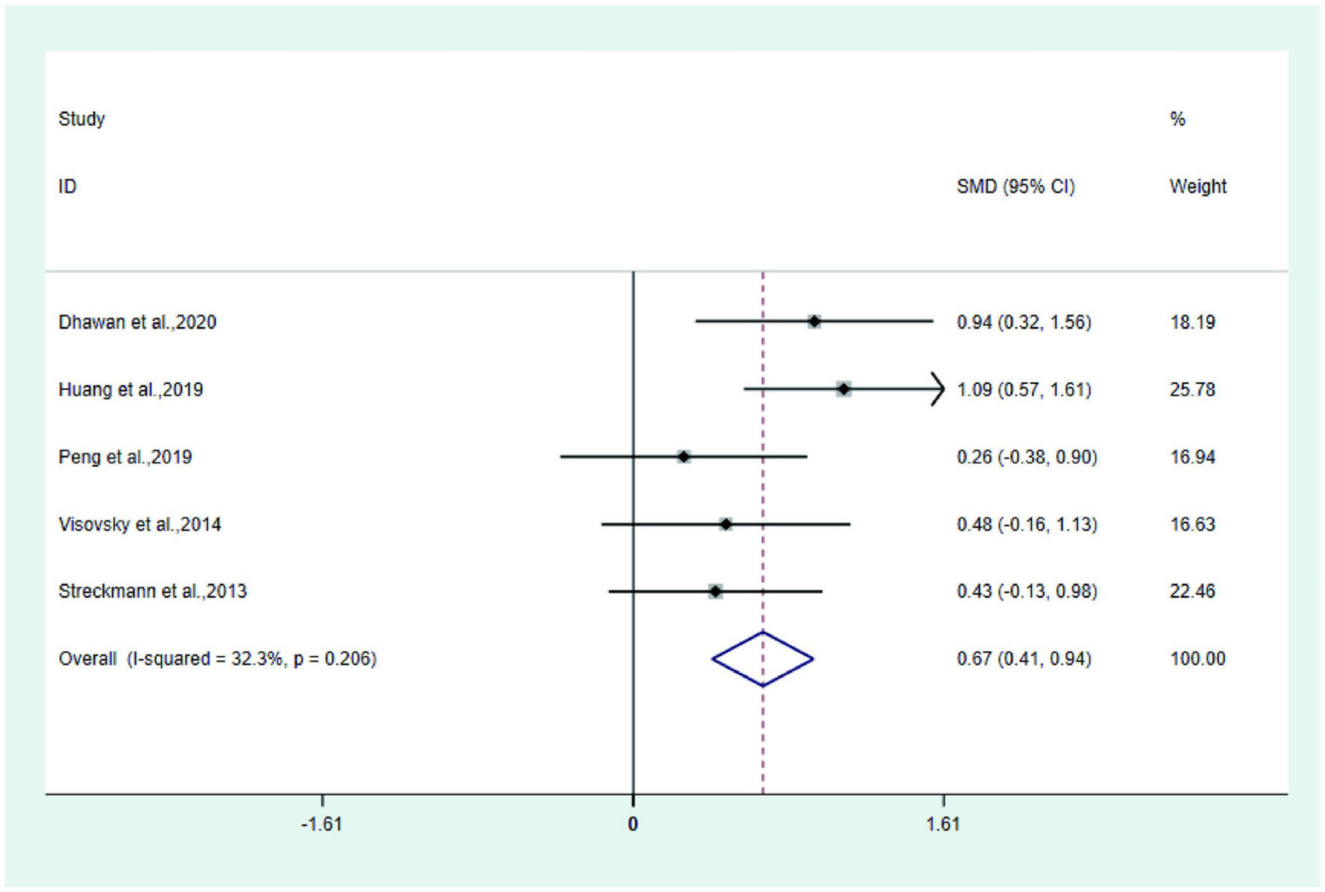
Forest plot of quality of life score.

①Total quality of life score Five studies (14, 22, 26, 30, 32) reported the total quality of life score in the test and control groups. Meta-analysis showed that the quality of life score of the test group was significantly higher than that of the control group (SMD: 0.67; 95% Cl: 0.41, 0.94; I^2^ = 32.3%, Figure 6).

**Figure 7:**
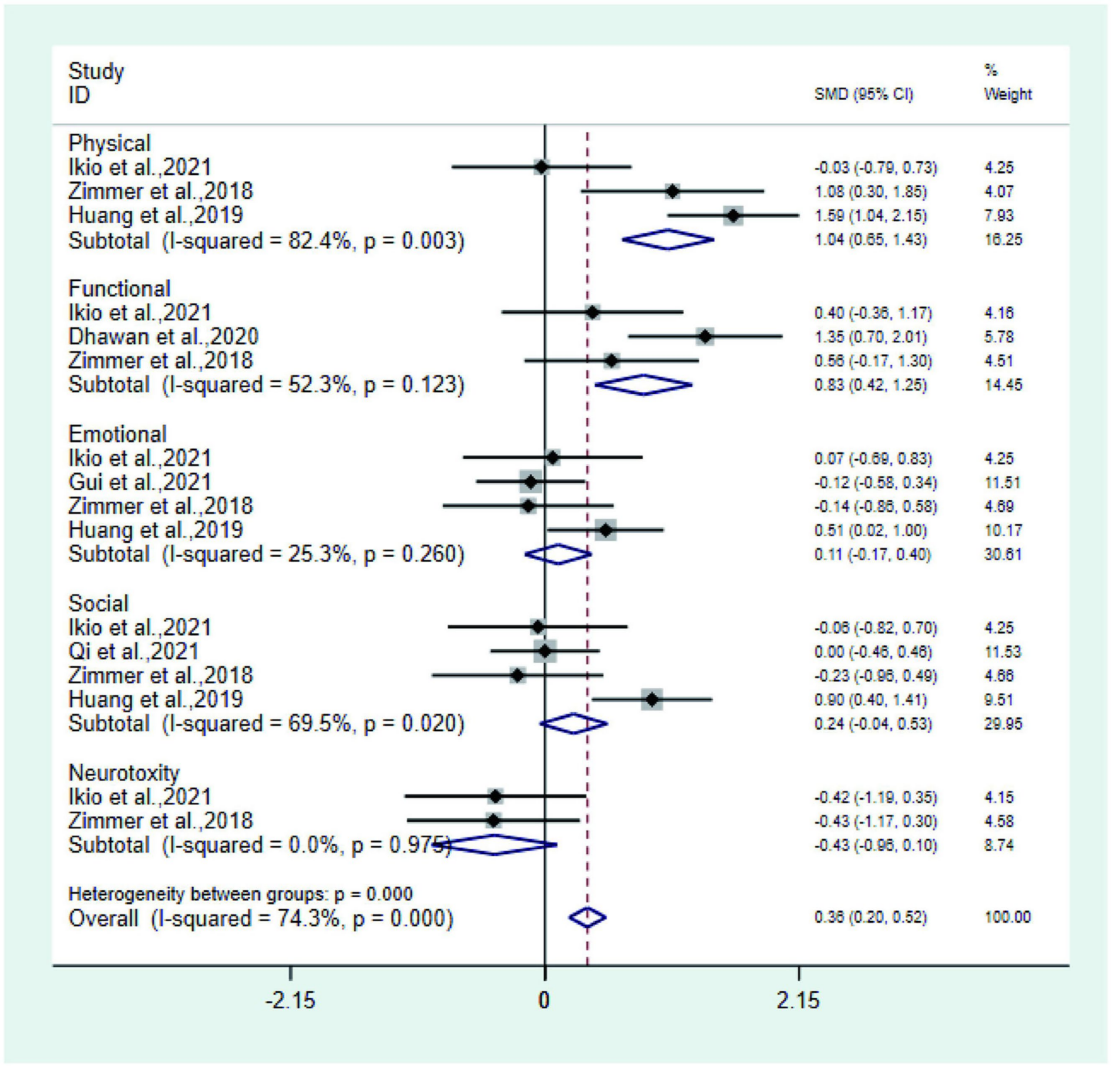
Forest plot of physical, functional, social, emotional, and neurotoxicity.

② Physical, functional, social, emotional and neurotoxicity studies (24, 26, 33) reported the physical of the test group and the control group, Meta-analysis showed that the physical of the test group was significantly higher than that of the control group (SMD: 1.04; 95% Cl: 0.65, 1.43; I^2^ = 82.4%); three studies (22, 24, 33) reported the functional of the test group and the control group, and Meta-analysis showed that the functional of the test group was significantly higher than that of the control group (SMD: 0.83; 95% Cl: 0.42, 1.25; I^2^ = 82.4%). The social, emotional, and neurotoxicity of the test group was not statistically significant compared with the control group (Figure 7).

#### 3.3.3 Pain

**Figure 8:**
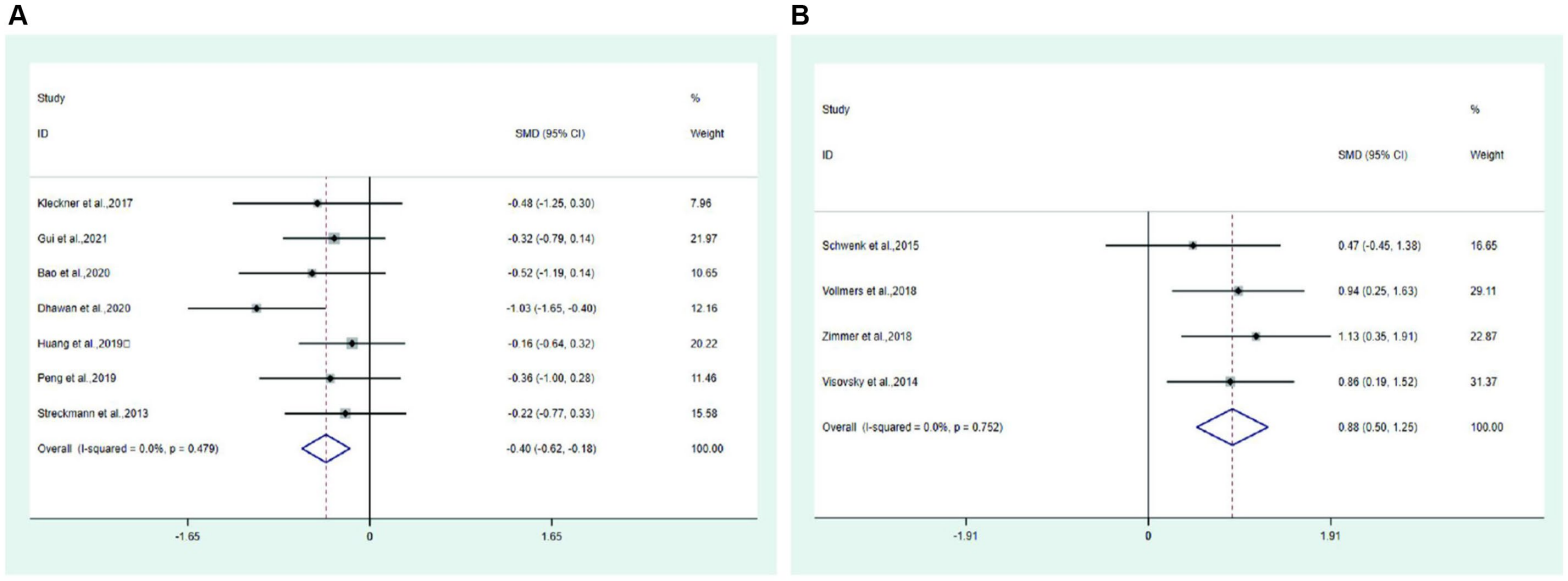
Forest plot. **(A)** Pain. **(B)** Balance.

Pain Seven studies (14, 21–23, 25, 26, 29, 32) reported pain in the test group and control group. Meta-analysis showed that the pain of the test group was significantly lower than that of the control group (SMD: −0.40; 95% Cl: −0.62, −0.18; I^2^ = 0.0%, Figure 8A).

#### 3.3.4 Balance

Balance Four studies (28, 30, 31, 33) reported balance in the test and control groups. Meta-analysis showed that the balance of the test group was significantly higher than that of the control group (SMD: 0.88; 95% Cl: 0.50, 1.25; I^2^ = 0.0%, Figure 8B).

#### 3.3.5 Fact/GOG-NTX

**Figure 9:**
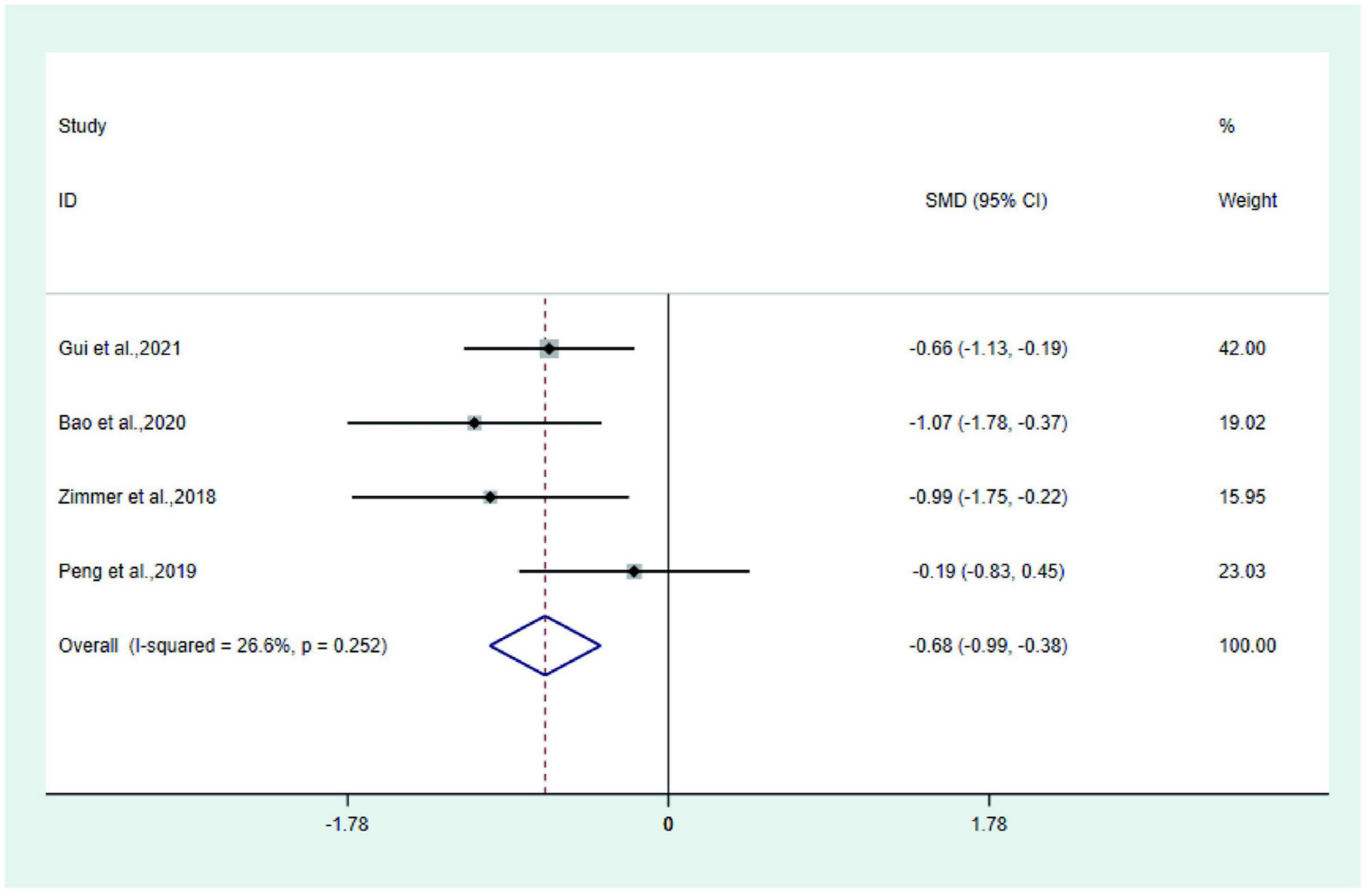
Forest plot of FACT/GOG-NTX.

Four studies (21, 23, 31, 33) reported FACT/GOG-NTX in the test and control groups. Meta-analysis showed that FACT/GOG-NTX in the test group was significantly lower than that in the control group (SMD: −0.68; 95% Cl: −0.99, −0.38; I^2^ = 26.6%, Figure 9).

### 3.4 Subgroup meta-analysis

Total symptom score has high heterogeneity, so we did subgroup meta-analysis to find the source of heterogeneity (Supplementary Figure 3). We performed subgroup meta-analysis of CIPN with different disease duration (2.1 m 3 m 4.01 m 10 m). The subgroup meta-analysis showed that excluding the 2.1 m (20), 10 m (22), the combined results of 3 m and 4.01 m were less heterogeneous (I^2^ = 0.0%). Combined with sensitivity analyses, it suggests that the two studies may be the source of high heterogeneity, and that disease duration affects the difference in treatment outcome of exercise therapy.

### 3.5 Publication bias

**Figure 10:**
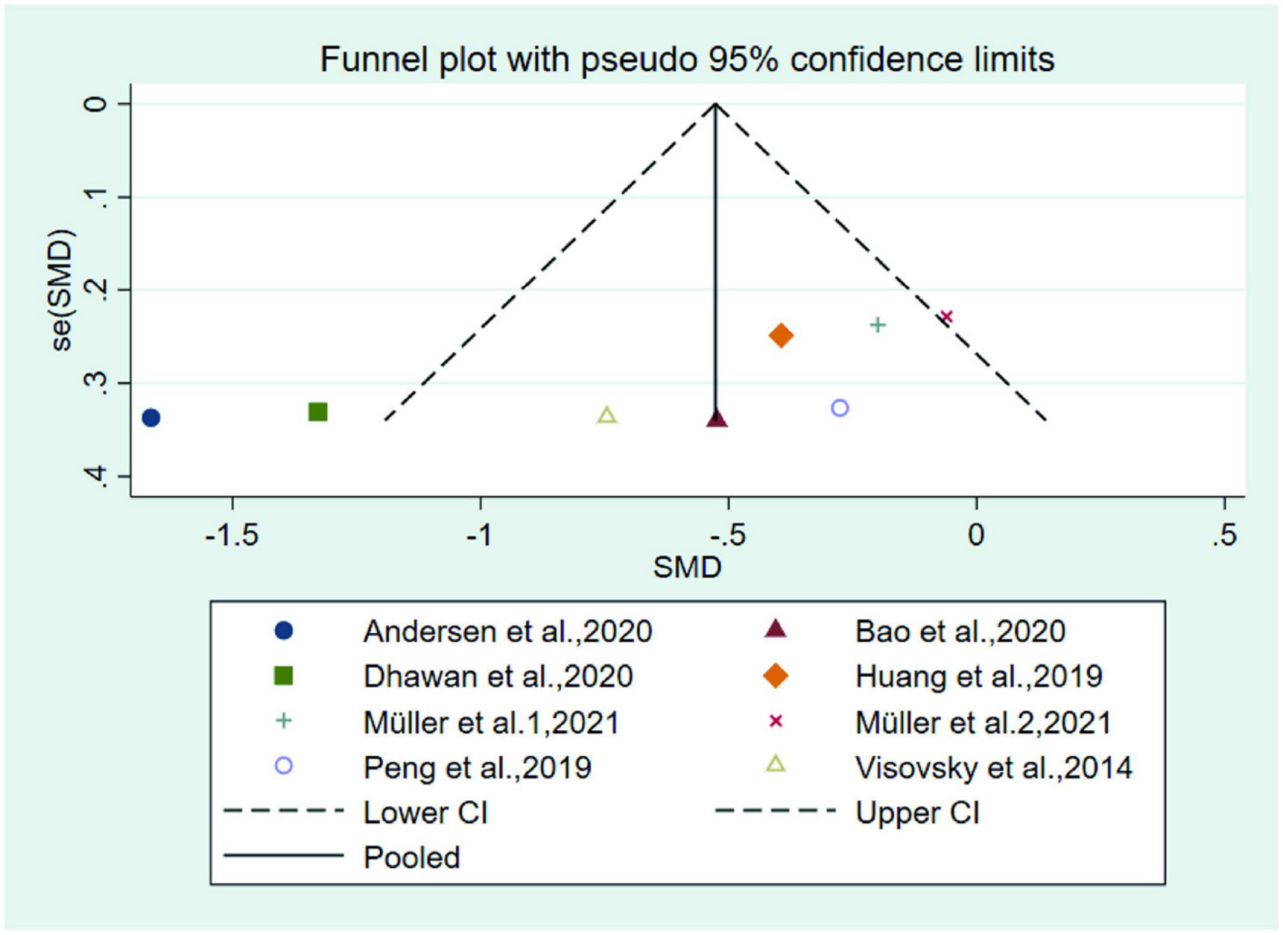
Funnel plot of the total symptom score.

Funnel plot of total symptom score (Figure 10) showed that there were two studies (20, 22) located outside the 95% CI, which may cause some publication bias. The reason for publication bias may be that 1. Higher frequency of interventions in these two test group than other studies; 2. Small sample size may cause some publication bias.

### 3.6 Certainty assessment

The GRADE protocol was used to assess the certainty of the evidence (Supplementary Table 1). Accordingly, studies investigating the effect of total symptom score was regarded as very low quality, respectively, due to the moderate heterogeneity between studies and publication bias and risk of bias. The evidence for Physical, functional, social, emotional, neurotoxicity and were graded as low due to inconsistencies or imprecision.

## 4 Discussion

Exercise therapy, as a simple and feasible non-pharmacological intervention, has initially achieved results in the prevention and treatment of CIPN, However, the scattered nature of the evidence and the controversial nature of the efficacy create confusion in the choice of physicians and patients, so the exact efficacy of exercise in improving CIPN is a question that deserves further exploration. Based on the results of 15 randomized controlled trials, this paper analyzes the peripheral neuropathy caused by exercise therapy after chemotherapy. Meta-analysis shows that the use of exercise therapy in CIPN patients is effective in reducing their own numbness, pain, improving the quality of life, reducing pain and improving balance.

Among the 15 randomized controlled studies we included, there was some variation in the frequency and duration of each exercise session, with five studies (14, 26, 28, 31, 33) were twice a week，three studies (24, 27, 30) were three times a week, one study (20) was four time a week, one study (29) was five times a week, four studies (21–23, 25) were once a day and one study (32) was once every other day.

The majority of each session lasted around 30–60 min. Although the frequency of intervention varied in each study, we found that the individualized training programs adopted in each of these studies, which were tailored to the different physical conditions of the patients, confirmed that exercise was effective in reducing the symptoms of treatment-induced neuropathy, while there was no increase in the severity of neuropathy symptoms after the exercise intervention. Of the studies we included, all were combined exercises, except for TingBao (21), peng (32), and Elizabeth (20) where the interventions were yoga and balance training alone, and neuro gliding. In addition to this, we found that receiving more physical activity and greater muscle bulk during exercise was also more likely to reduce the symptoms of CIPN. The exception to this is the elderly, who because of their specific abilities, have an increased fear of physical injury and falls due to excessive exercise, so the results of the Michael (28) study, which discussed a specific training program for the elderly, suggest that providing an adequate dose of exercise to elderly patients can also significantly reduce the symptoms of CIPN and reduce the risk of injury from falls. All of the exercise interventions studied were performed after chemotherapy, although the frequency and severity of CIPN subsided gradually with the cessation of chemotherapy. However, all patients showed varying degrees of remission after exercise. Also, the result of meta-analysis showed that exercise in the test group reduced the symptoms and severity of CIPN compared to the control group.

The human nervous system is divided into the central nervous system and peripheral nervous system. Because the central nervous system has the protection of blood–brain barrier, chemotherapeutic drugs are not easy to enter, on the contrary, the peripheral nervous system lacks the corresponding protection, so chemotherapeutic drugs easily enter and destroy the peripheral nerve cells. CIPN symptoms are caused precisely by the action of certain chemotherapy drugs on the body’s peripheral nerves, causing patients to experience damage to sensory, motor and autonomic nerves. Pain receptor sensitization is an abnormal lesion of sensory nerves, and excessive release of inflammatory factors can lead to receptor sensitization, and chemotherapeutic drugs have been reported to up-regulate serum inflammatory factor levels (35) (IL-6, IL-8, IL-1P, TNF-a) in patients, thereby aggravating the severity of symptoms in patients. Exercise can reduce inflammation and oxidative stress in the body (36) by promoting the release of anti-inflammatory cytokines (37) and reducing oxidative markers (38), which can lead to diminished sensory symptoms. Meanwhile, the imbalance between neuronal apoptosis and the metabolic capacity of the nervous system is an important condition for the development of CIPN, and platinum-based compounds can damage the dorsal root ganglia (39), destroy the structure of DNA, and induce neuronal apoptosis (40). While exercise increases neuronal metabolism (40) and promotes secretion (41) of nerve growth factor, neurotrophins, etc., It is through this mechanism that damaged nerve fibers are regenerated and peripheral nervous system plasticity (42) is increased to achieve symptom relief in the balance training we incorporate.

In the studies we included, it can be shown that exercise therapy can improve the symptoms of CIPN. In a multi-center Randomized controlled trial, 170 cancer patients with CIPN were treated with moderate intensity exercise therapy for six weeks, and the symptoms of CIPN such as numbness of hands and feet, burning sensation were significantly alleviated. Duregon’s systematic review of 5 RCTs also confirmed the effectiveness of exercise therapy in preventing and improving CIPN. Our findings fit with the Lin et al. (43) which included five studies examining the effects of different exercise programs (general physical activity, Tai Chi, dance, yoga, and Pilates), all of which affirmed the effectiveness of exercise therapy interventions for CIPN, but the strength of this paper lies in the inclusion of more studies and the systematic dissection of CIPN symptoms, quality of life, and sense of balance. Further evidence of the supportive nature of exercise therapy interventions for CIPN.

The imbalance between neuronal apoptosis and the metabolic capacity of the nervous system is an important condition for the development of CIPN, and platinum-based compounds can damage the dorsal root ganglia (39), destroy the structure of DNA, and induce neuronal apoptosis (40). While exercise increases neuronal metabolism (40) and promotes secretion (41) of nerve growth factor, neurotrophins, etc., It is through this mechanism that damaged nerve fibers are regenerated and peripheral nervous system plasticity (42) is increased to achieve symptom relief in the balance training we incorporate. In an animal study (44), studies mimicking traumatic peripheral nerve injury in humans and mice have shown that exercise relieves neuropathy through various neurophysiological mechanisms in the peripheral nervous system, central nervous system. CIPN can affect the psychological status of cancer patients (45), aggravate anxiety and depression (46) and other adverse emotions, exercise can stimulate the brain to produce endorphin (47), regulate hormone levels (48), so that exercise can obtain a pleasant and comfortable experience. This reduces the psychological burden. In the studies we included, the interventions were delivered through long cycles of exercise, which consistently enhanced muscle oxygenation (49), improving the body’s activity endurance, enhancing its physical status and reducing pain, thus reducing the impact of CIPN on patients’ daily activities and consequently improving their quality of life.

In addition, social, emotional, and neurotoxicity in quality of life were not statistically significant in this meta-analysis. The reason for this analysis may be due to insufficient statistics included, but combining the results of other clinical studies, exercise therapy intervention can significantly improve the social, emotional and neurotoxicity in the quality of life of patients, which is also consistent with a large number of exercise oncology studies (50).

Third, cancer patients with CIPN mostly experience motor and sensory disturbance symptoms such as lower limb dysesthesia and fatigue, while sensory disturbance makes the sensory system unable to provide information to control cutaneous sensory nerve receptors (34), resulting in the inability of the human body to complete precise movements, and these abnormalities can affect the patient ‘s balance function and increase the risk of falls. Intervention all through exercise induces neurons, muscles, and metabolic systems (34) of some adaptive processes, resulting in better stimulated postural control (51, 52), enhancing vestibular organ stability (53), and improving body balance. Our findings fit with the Lin et al. (43), which included five studies examining the effects of different exercise programs (general physical activity, Tai Chi, dance, yoga, and Pilates), all of which affirmed the effectiveness of exercise therapy interventions for CIPN, but the strength of this paper lies in the inclusion of more studies and the systematic dissection of CIPN symptoms, quality of life, and sense of balance. Further evidence of the supportive nature of exercise therapy interventions for CIPN.

The limitations of this study are: 1. the duration and frequency of treatment are different every week, and there are differences between onset and start of treatment; 2. there is a lack of reporting in adverse reactions in the included studies; 3. due to the non-uniform diagnostic criteria for CIPN, there may be some bias in the results.

In conclusion, exercise therapy can be effective in treating CIPN by improving symptom score (total symptom score, numbness, tingling), quality of life score (total score, physical function), pain, balance, and FACT/GOG-NTX questionnaires. The findings are consistent with the current results (14, 20–33)on this subject, but the most appropriate exercise therapy for each age group of patients can be refined in the future, and the findings of this study still need to be refined and validated by more high-quality, multicenter, large-sample RCTs in the future to further explore the application of exercise therapy in patients with CIPN and its long-term effects.

## Author contributions

YH: conceptualization. YH, ML, and JX: acquisition of data. YH and TT: analysis and/or interpretation of data. YH, TT, and LL: drafting the manuscript. ML and JX: revising the manuscript critically for important intellectual content and supervision. YH, TT, ZY, YD and GL: data validation. All authors contributed to the article and approved the submitted version.

## Supplementary material

The Supplementary material for this article can be found online at:

https://www.frontiersin.org/articles/10.3389/fneur.2023.1252259/full#supplementary-material
