# Corrigendum: Exercise for reducing chemotherapy-induced peripheral neuropathy: a systematic review and meta-analysis of randomized controlled trials

**DOI:** 10.3389/fneur.2024.1378461

**Published:** 2024-03-07

**Authors:** Yingjie Huang, Tian Tan, Lu Liu, Zijian Yan, Yuexia Deng, Guangyao Li, Min Li, Jia Xiong

**Affiliations:** ^1^Guangzhou University of Chinese Medicine, Guangzhou, China; ^2^Clinical Medical College of Acupuncture Moxibustion and Rehabilitation, Guangzhou University of Chinese Medicine, Guangzhou, China; ^3^The First Clinical College of Guangzhou University of Chinese Medicine, Guangzhou, China; ^4^Southern Theater General Hospital, Guangzhou, China; ^5^Department of Traditional Chinese Medicine, Guangdong Provincial Key Laboratory of Major Obstetric Diseases, Guangdong Provincial Clinical Research Center for Obstetrics and Gynecology, The Third Affiliated Hospital of Guangzhou Medical University, Guangzhou, China; ^6^Nanchang Hongdu Hospital of Traditional Chinese Medicine, Nanchang, Jiangxi Province, China

**Keywords:** exercise therapy, chemotherapy-induced peripheral neuropathy, CIPN, efficacy, meta-analysis

In the published article, there was an error in affiliation 2. Instead of “Department of Traditional Chinese Medicine, Guangdong Provincial Key Laboratory of Major Obstetric Diseases, Guangdong Provincial Clinical Research Center for Obstetrics and Gynecology, The Third Affiliated Hospital of Guangzhou Medical University, Guangzhou, China”, it should be “Clinical Medical College of Acupuncture Moxibustion and Rehabilitation, Guangzhou University of Chinese Medicine, Guangzhou, China”.

In the published article, there was an error regarding the affiliations for Guangyao Li. Instead of affiliation 2, they should have “Department of Traditional Chinese Medicine, Guangdong Provincial Key Laboratory of Major Obstetric Diseases, Guangdong Provincial Clinical Research Center for Obstetrics and Gynecology, The Third Affiliated Hospital of Guangzhou Medical University, Guangzhou, China”.

The authors apologize for this error and state that this does not change the scientific conclusions of the article in any way. The original article has been updated.

