# Corrigendum: Exercise for reducing chemotherapy-induced peripheral neuropathy: a systematic review and meta-analysis of randomized controlled trials

**DOI:** 10.3389/fneur.2024.1460992

**Published:** 2024-08-01

**Authors:** Yingjie Huang, Tian Tan, Lu Liu, Zijian Yan, Yuexia Deng, Guangyao Li, Min Li, Jia Xiong

**Affiliations:** ^1^Guangzhou University of Chinese Medicine, Guangzhou, China; ^2^Clinical Medical College of Acupuncture Moxibustion and Rehabilitation, Guangzhou University of Chinese Medicine, Guangzhou, China; ^3^The First Clinical College of Guangzhou University of Chinese Medicine, Guangzhou, China; ^4^Southern Theater General Hospital, Guangzhou, China; ^5^Department of Traditional Chinese Medicine, Guangdong Provincial Key Laboratory ofMajor Obstetric Diseases, Guangdong Provincial Clinical Research Center for Obstetrics and Gynecology, The Third Affiliated Hospital of Guangzhou Medical University, Guangzhou, China; ^6^Nanchang Hongdu Hospital of Traditional Chinese Medicine, Nanchang, Jiangxi Province, China

**Keywords:** exercise therapy, chemotherapy-induced peripheral neuropathy, CIPN, efficacy, meta-analysis

In the published article, there was an error in [Figures 3–10 and Table 1] as published. [The figures and table incorrectly list studies with first names of the authors, instead of last names]. The corrected [Figures 3–10 and Table 1] and their captions appear below.

**Figure 3:**
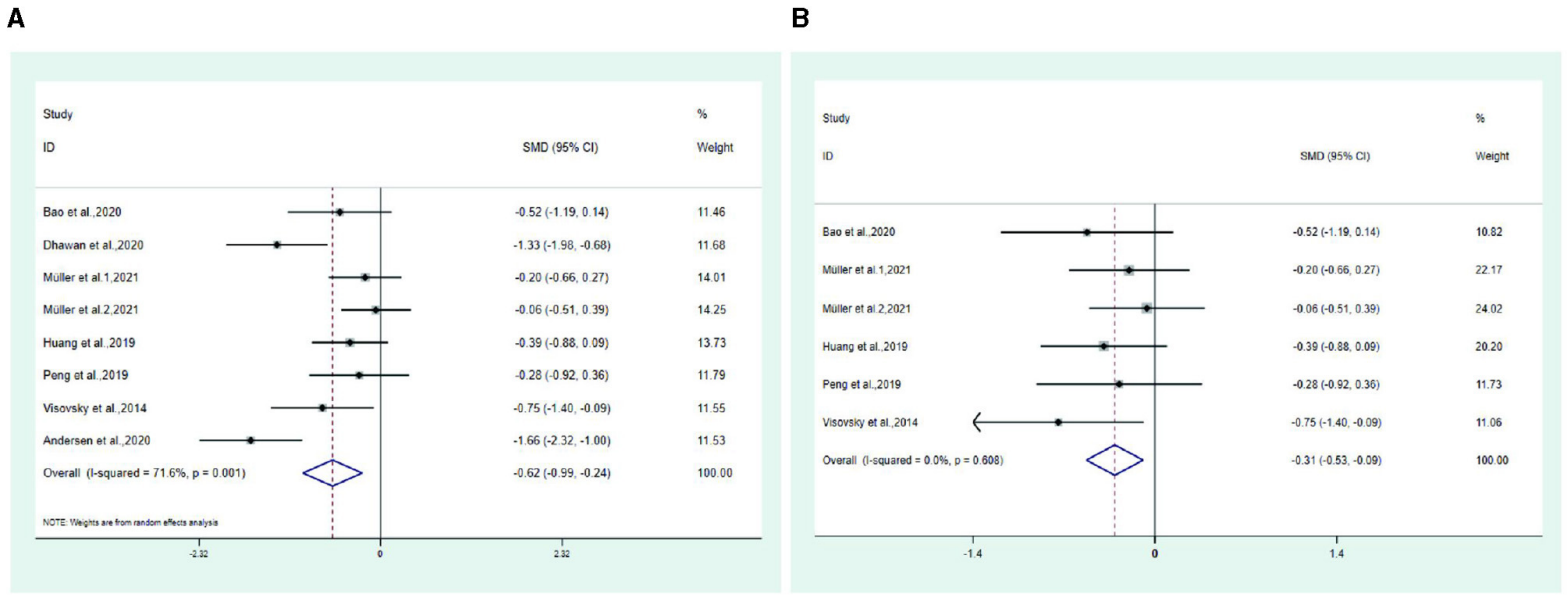
Forest plot. **(A)** Total symptom score. **(B)** Total symptom score after excluding two trail.

**Figure 4:**
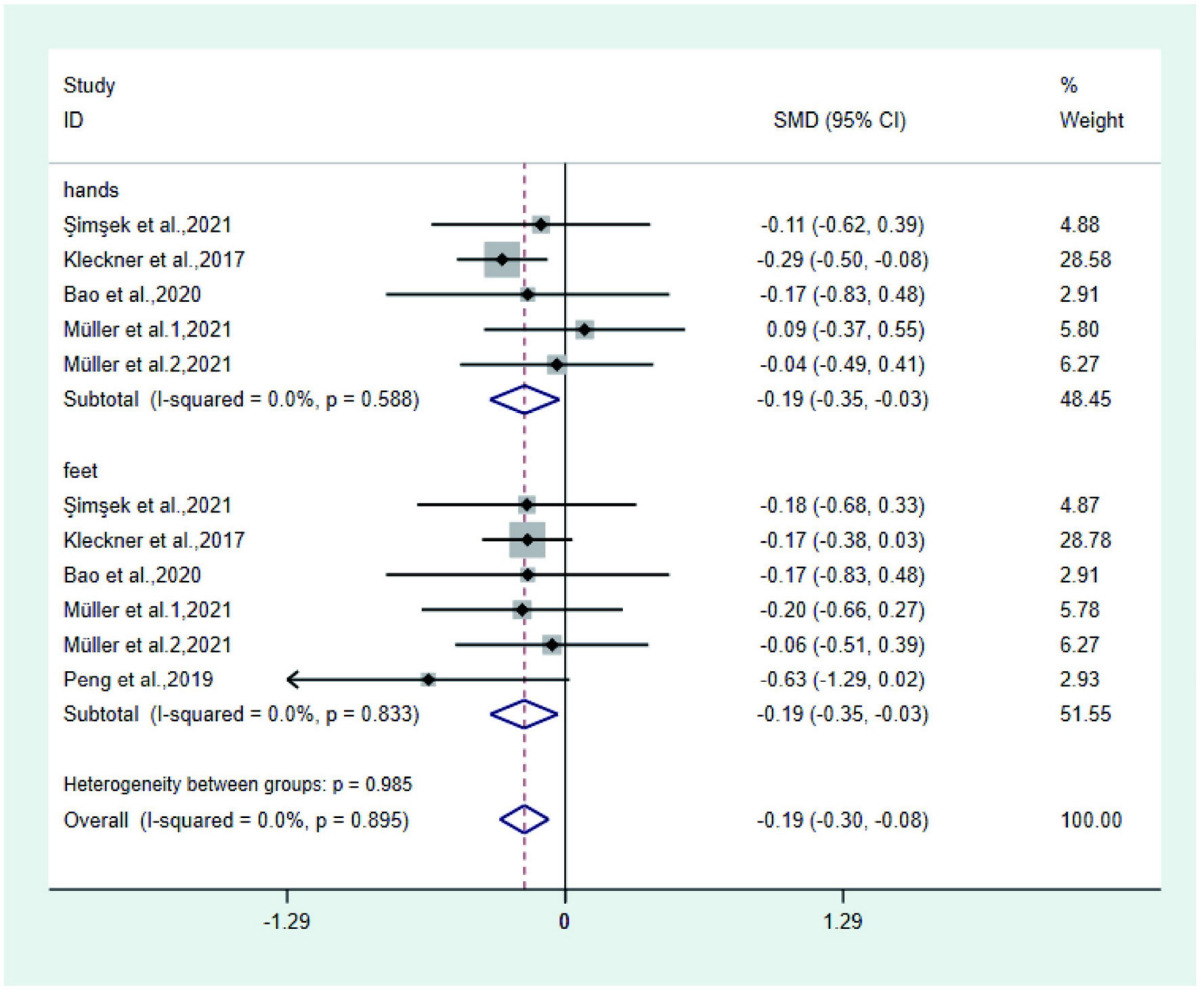
Forest plot of numbness.

**Figure 5:**
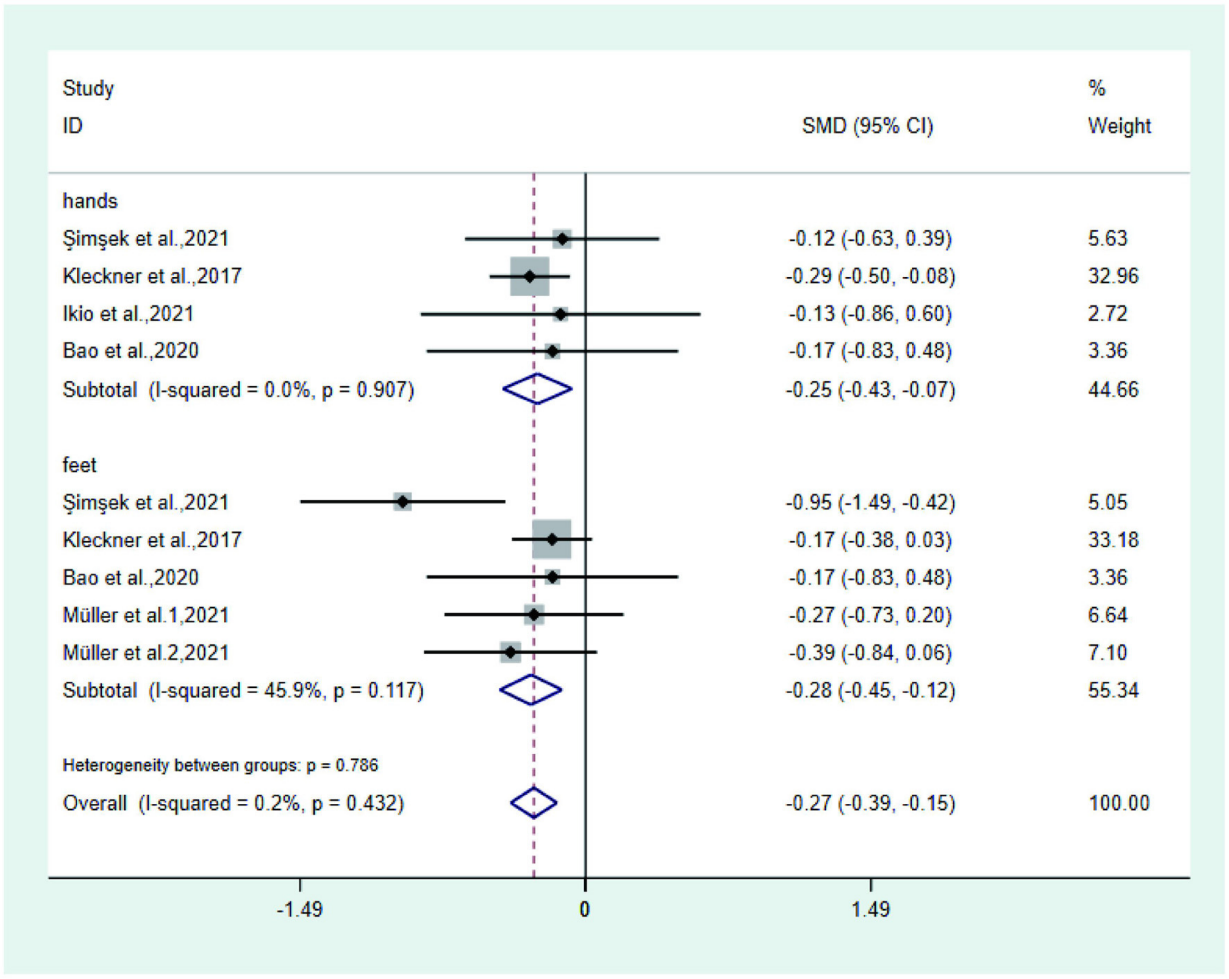
Forest plot of tingling.

**Figure 6:**
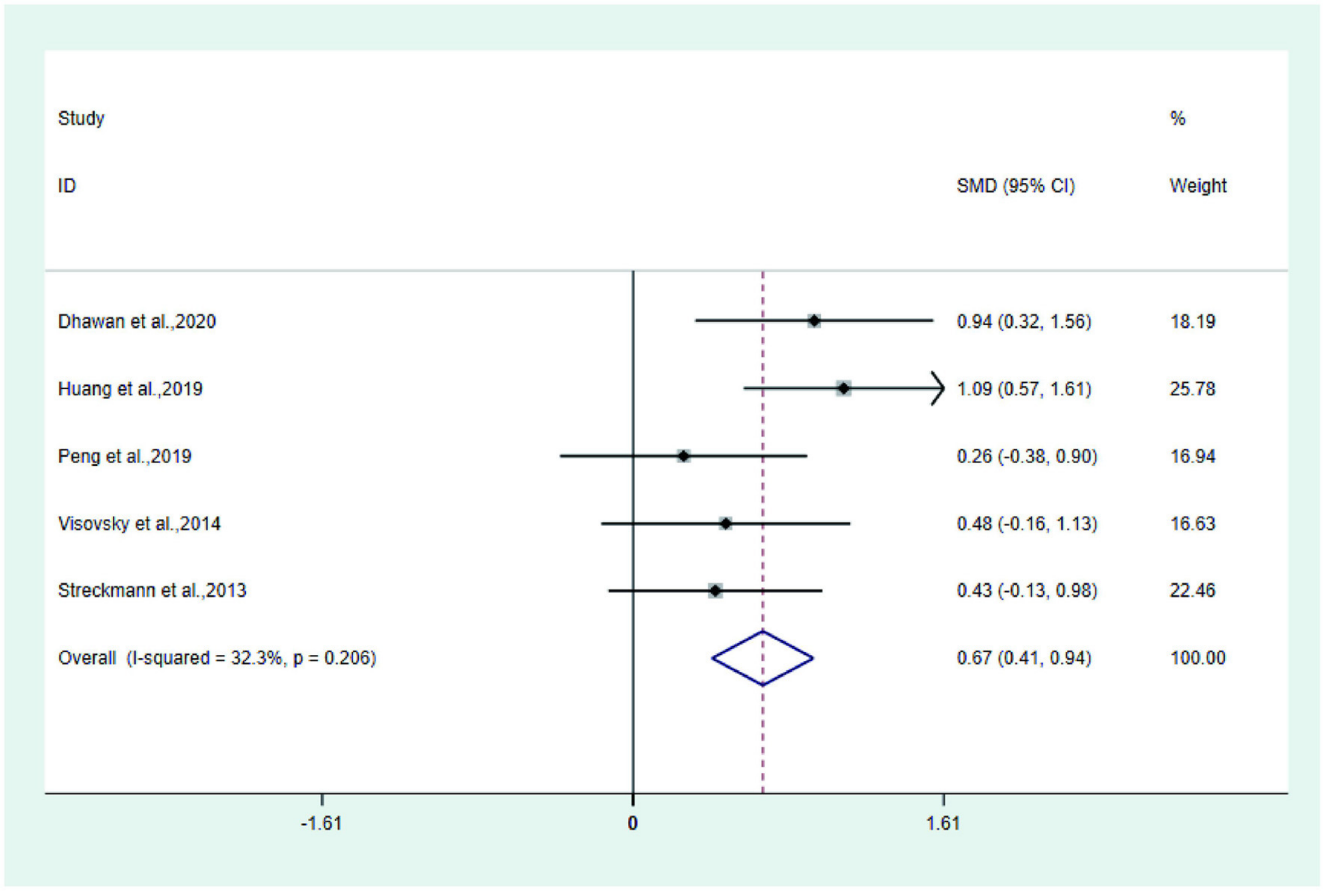
Forest plot of quality of life score.

**Figure 7:**
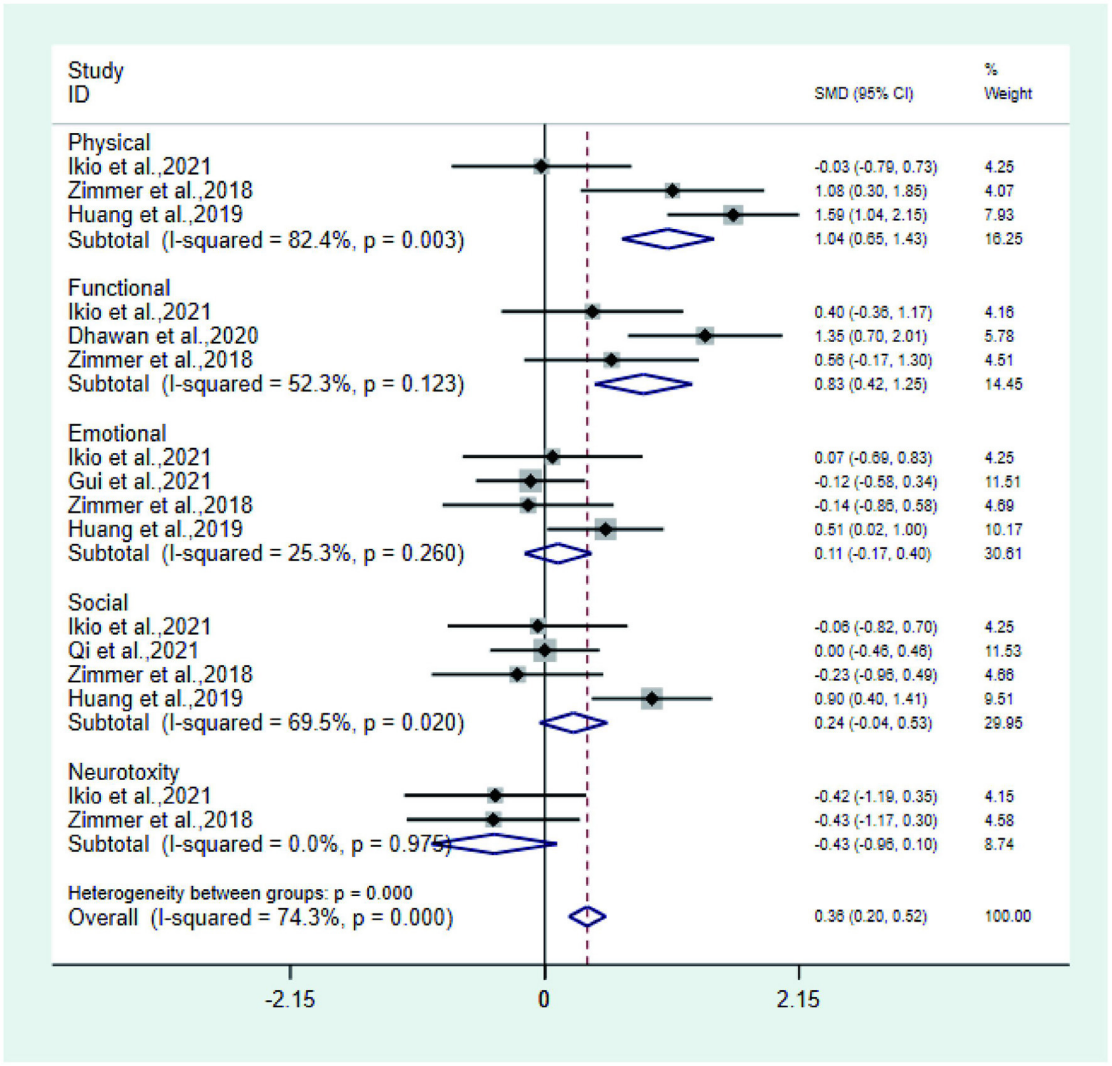
Forest plot of physical, functional, social, emotional, and neurotoxicity.

**Figure 8:**
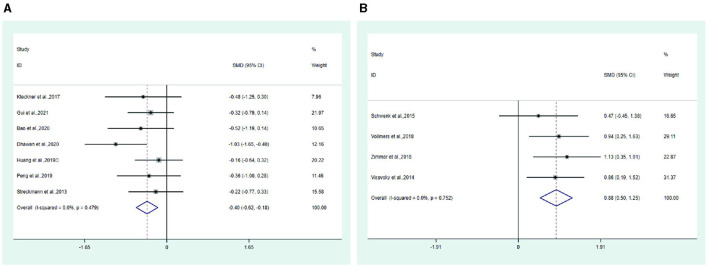
Forest plot. **(A)** Pain. **(B)** Balance.

**Figure 9:**
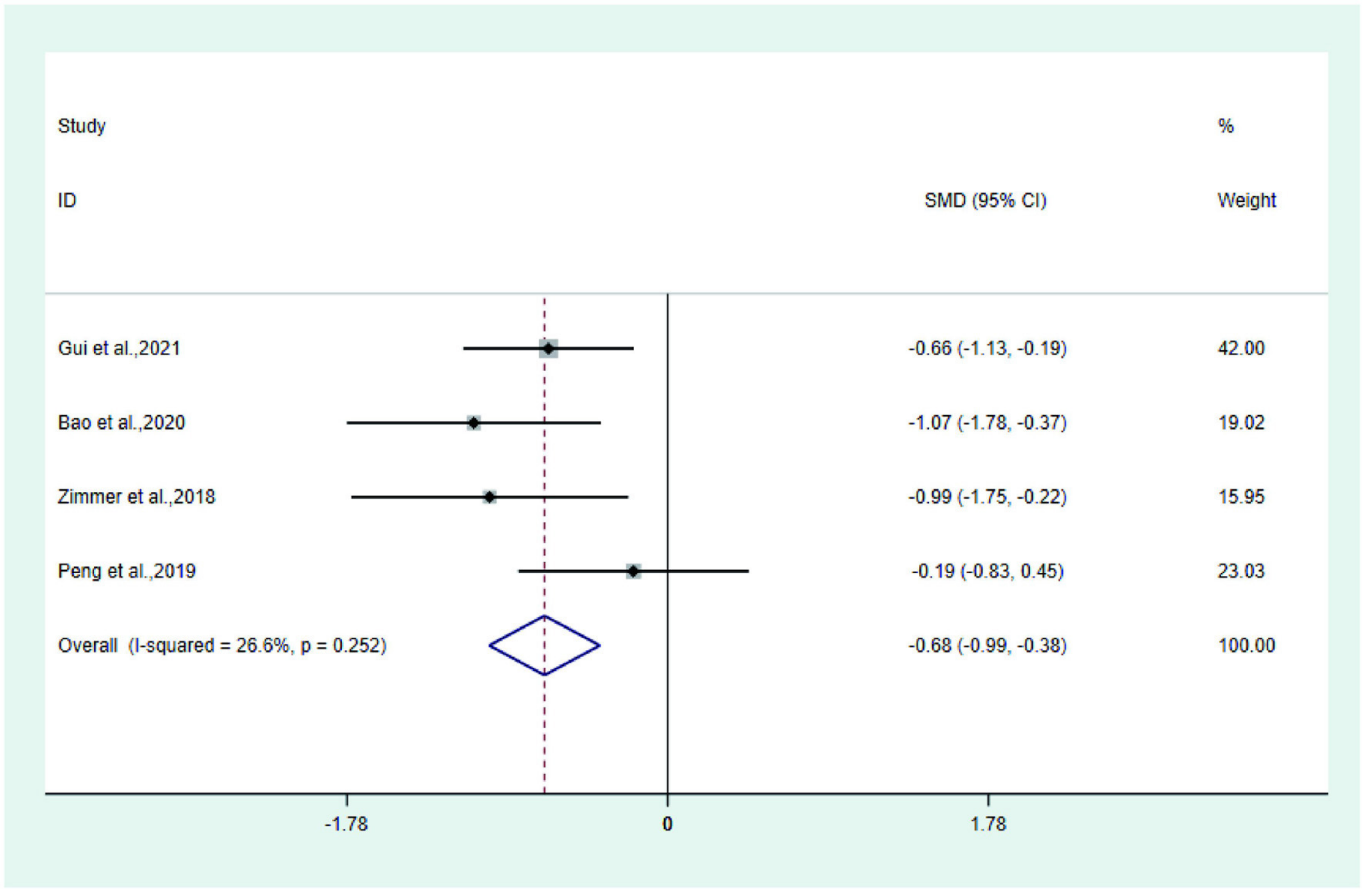
Forest plot of FACT/GOG-NTX.

**Figure 10:**
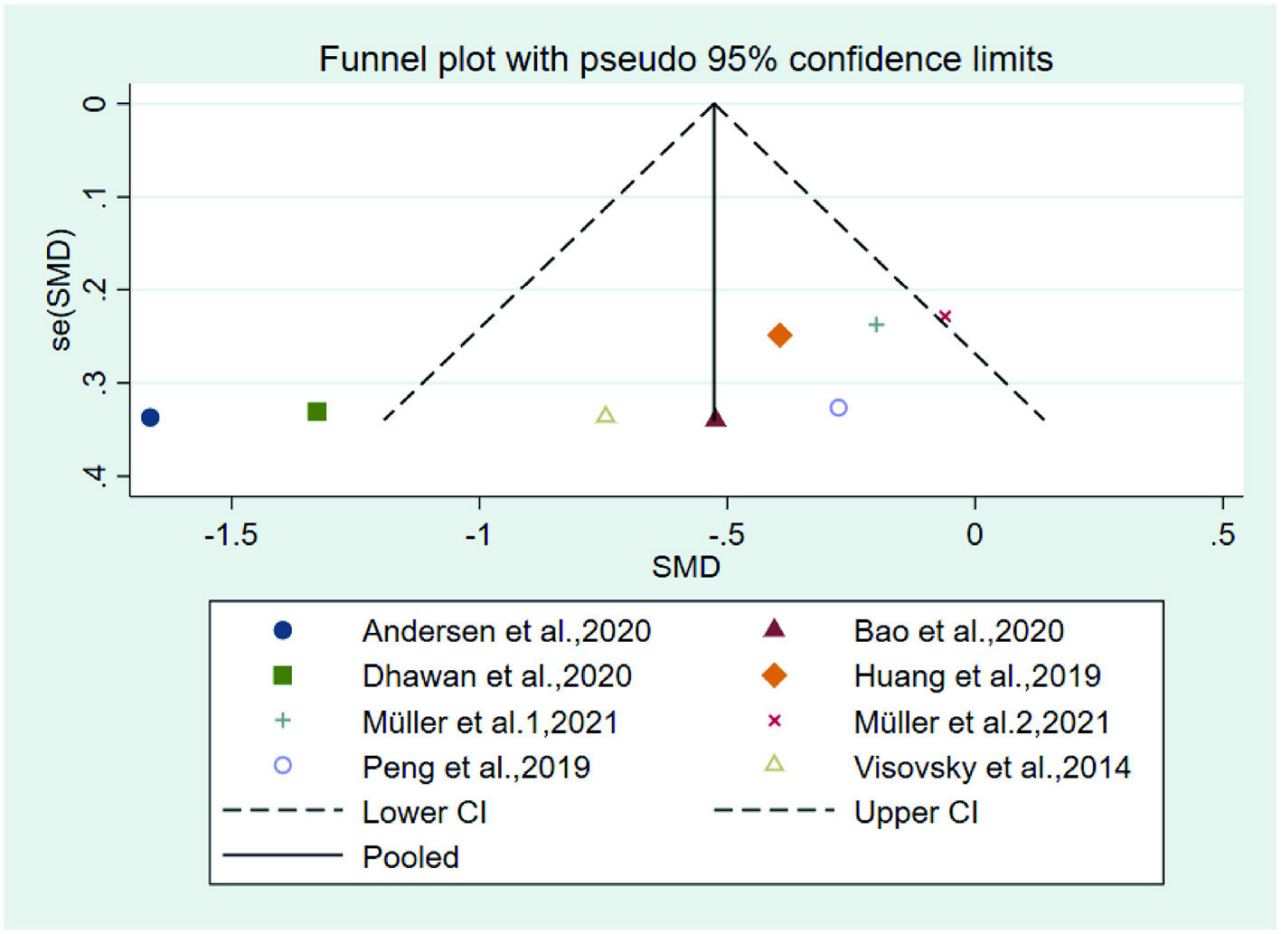
Funnel plot of the total symptom score.

**Table 1:** The basic characteristics of the included studies.

**Trail**	**Country**	**Sample size (T/C)**	**Age (y), Mean** ±**SD or median (Range)**		**Duration/W**		**T**	**C**	**Main outcomes**	**Frequency**	**Follow-uptime/week**	**Cancer**	**Chemotherapy**
			**T**	**C**	**T**	**C**							
Simşek et al. (29)	Turkey	30, 30/30	>18y	>8y	NA	NA	Stretching exercise + balance exercise	Chemotherapy + usual care		5 times/ Week	12	Breast cancer	Taxane
Kleckner et al. (25)	USA	170/185	55.6 ± 11.8	55.9 ± 9.7	12.1 ± 60.9	5.7 ± 10.0	Strength training	Chemotherapy		60 min, qd	6	Breast/ Lymphoma/ Colon/ Lung cancer	Taxane-, platinum-, or vinca alkaloid
Ikio et al. (24)	Japan	19/20	69 (60–89)	64 (57–87)	5 (2–111)	6 (2–93)	Strength training	Chemotherapy		30 min, tiw	NA	Hematological malignancy/ Gastrointestinal cancer	Vinca alkaloids, taxanes, platinum compounds and proteasome inhibitors
Gui et al. (23)	China	51/28	50 ± 8	52 ± 7	12 (23.5)	6 (21.4)	Sensory exercise	Chemotherapy		qd	2	Digestive malignancies	Oxaliplatin
Bao et al. (21)	USA	21/20	60.0 (35.5, 77.9)	62.3 (42.4, 79.0)	3.1 (0.5, 10.4)	3.7 (0.9, 15.3)	Yoga	Chemotherapy + usual care		60 min, qd	8	Breast/ Uterine/ Ovarian cancer	Taxane-, platinum-, or vinca alkaloid
Dhawan et al. (22)	India	22/23	50.5 ± 7.9	52.5 ± 6.6	10.2 ± 7.8	9.8 ± 8.6	Strength training	Chemotherapy		30 min, qd	10	Ovarian/ Cervical/ Lung cancer	Paclitaxel, carboplatin
Müller et al. (27)	Germany	49, 57/57	51.7 ± 10.8/53.4 ± 11.7	54.5 ± 11.9	22.0 ± 9.3/23.5 ± 8.9	23.0 ± 9.4	Sensory exercise/ strength training	Chemotherapy		35min, tiw	NA	NA	Taxanes, platinum derivatives, vinca alkaloids
Schwenk et al. (28)	USA	11./11	68.73 ± 8.72	71.82 ± 8.85	49.91 ± 44.11	44.63 ± 56.78	Balance exercise	Chemotherapy + health education		45 min, biw	4	NA	NA
Vollmers et al. (31)	Germany	17/19	48.56 ± 11.94	52.39 ± 10.14	12	12	Sensory exercise	Chemotherapy + health education		60 min, biw	12	Breast cancer	Taxane
Zimmer et al. (33)	Germany	17/13	68.53 (50–81)	70.00 (50–81)	27.94 ± 24.557	24.38 ± 19.692	Strength training + balance exercise	Chemotherapy + health education		60 min, biw	8	Colon cancer	Bevacizumab, regorafenib, trastuzumab
Huang et al. (26)	China	32/34	54.56 ± 9.4	57.65 ± 9.8	4.66 ± 1.10	4.79 ± 1.06	Aerobic exercise	Chemotherapy + usual care		30 min, biw	6	Ovarian cancer	Taxane-, platinum-, or vinca alkaloid
Peng et al. (32)	China	23/23	53.17 ± 9.29	52.15 ± 7.25	NA	NA	Balance exercise	Chemotherapy + usual care		15–30 min, tiw	12	Breast/ Lymphoma cancer/ Myeloma	Taxanes, platinum, vincristine, bortezomib, thalidomide
Visovsky et al. (30)	USA	19/19	48.8 (24–65)	48.8 (24–65)	NA	NA	Aerobic exercise + strength training	Chemotherapy + health education		30 min/ time, 5–7 times/ Week	12	Breast cancer	Taxane
Streckmann et al. (50)	Germany	28/28	44 (20–67)	48 (19–73)	NA	NA	Aerobic exercise + strength training	Chemotherapy		biw	36	Lymphoma cancer	Taxanes, platinum, vincristine, bortezomib, thalidomide
Andersen et al. (20)	Canada	22/26	56.3 ± 9.9	53 ± 10.3	NA	NA	Stretching exercise	Chemotherapy + usual care		4 times/ Week	6	Breast cancer	Taxane

T, trial group; C, control group; NA, not reported; symptom score (total symptom score, numbness, and tingling); quality of life score (total score, physical, functional, social, emotional, and neurotoxicity); pain; balance; functional assessment of neurotoxicity (FACT/GOG-NTX); qd, once a day; biw, 2 times/week; tiw, 3 times/week.

In the published article, there was an error in [Supplementary Figures 1–3]. [The supplementary figures incorrectly list studies with first names of the authors, instead of last names]. The correct material statement published in online.

The authors apologize for this error and state that this does not change the scientific conclusions of the article in any way. The original article has been updated.

