# Supplementary material for: Exercise for reducing chemotherapy-induced peripheral neuropathy: a systematic review and meta-analysis of randomized controlled trials
Source: Front Neurol. 2024 Jan 12;14:1252259. doi: 10.3389/fneur.2023.1252259 (PMC10813204; doi:10.3389/fneur.2023.1252259)
Supplement: Supplementary file 1 [file Table_1.docx]

Supplementary Table 1 GRADE evidence profile: exercise for CIPN

| Outcomes | Number of studies | Design |  |  | Quality assessment |  |  | Number of patients |  | Effect | Quality |
| --- | --- | --- | --- | --- | --- | --- | --- | --- | --- | --- | --- |
|  |  |  | Risk of bias | Inconsistency | Indirectness | Imprecision | Other considerations | Test group | Control group | SMD(95%CI) |  |
| Total symptom score | 7 | Randomized trials | Serious | Serious | No serious indirectness | No serious imprecision | Reporting bias 0.024 | 219 | 177 | -0.62(-0.99,-0.24) | Very low |
| Numbness |  | Randomized trials | Serious | No serious inconsistency | No serious indirectness | No serious imprecision | None | 323 | 291 | -0.19(-0.30,-0.08) | Moderate |
| Tingling |  | Randomized trials | Serious | No serious inconsistency | No serious indirectness | No serious imprecision | None | 323 | 291 | -0.27(-0.39,-0.15) | Moderate |
| Total quality of life score |  | Randomized trials | Serious | No serious inconsistency | No serious indirectness | No serious imprecision | None | 117 | 121 | 0.67(0.41,0.94) | Moderate |
| Physical, functional, social, emotional and neurotoxicity |  | Randomized trials | Serious | Serious | No serious indirectness | No serious imprecision | None | 134 | 113 | 0.36(0.20,0.52) | Low |
| Pain |  | Randomized trials | Serious | No serious inconsistency | No serious indirectness | No serious imprecision | None | 178 | 164 | -0.40(-0.62,-0.18) | Moderate |
| Balance |  | Randomized trials | Serious | No serious inconsistency | No serious indirectness | Serious | None | 62 | 61 | 0.88(0.50,1.25) | Low |
| FACT/GOG-NTX |  | Randomized trials | Serious | No serious inconsistency | No serious indirectness | No serious imprecision | None | 103 | 80 | -0.68(-0.99,-0.38) | Moderate |
