# Supplementary material for: Exercise for reducing chemotherapy-induced peripheral neuropathy: a systematic review and meta-analysis of randomized controlled trials
Source: Front Neurol. 2024 Jan 12;14:1252259. doi: 10.3389/fneur.2023.1252259 (PMC10813204; doi:10.3389/fneur.2023.1252259)
Supplement: Supplementary file 2 [file Data_Sheet_1.docx]

Supplementary Material

**Supplementary Figure 1.** Risk of bias assessment for each included study in the review.

**Supplementary Figure 2.** Sensitivity analysis of the total symptom score.

**Supplementary Figure 3.** The subgroup meta-analysis of total symptom score.


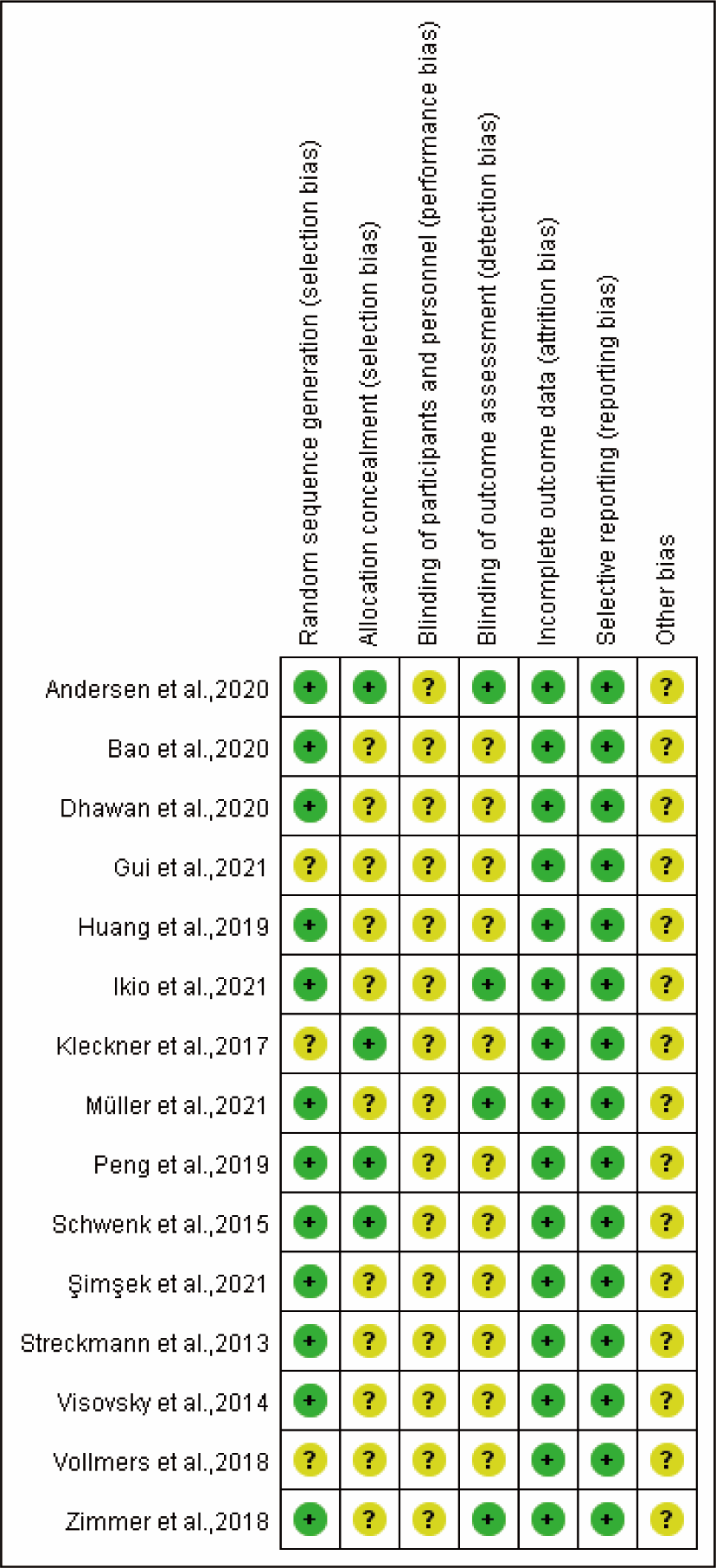


**Supplementary Figure 1.** Risk of bias assessment for each included study in the review.


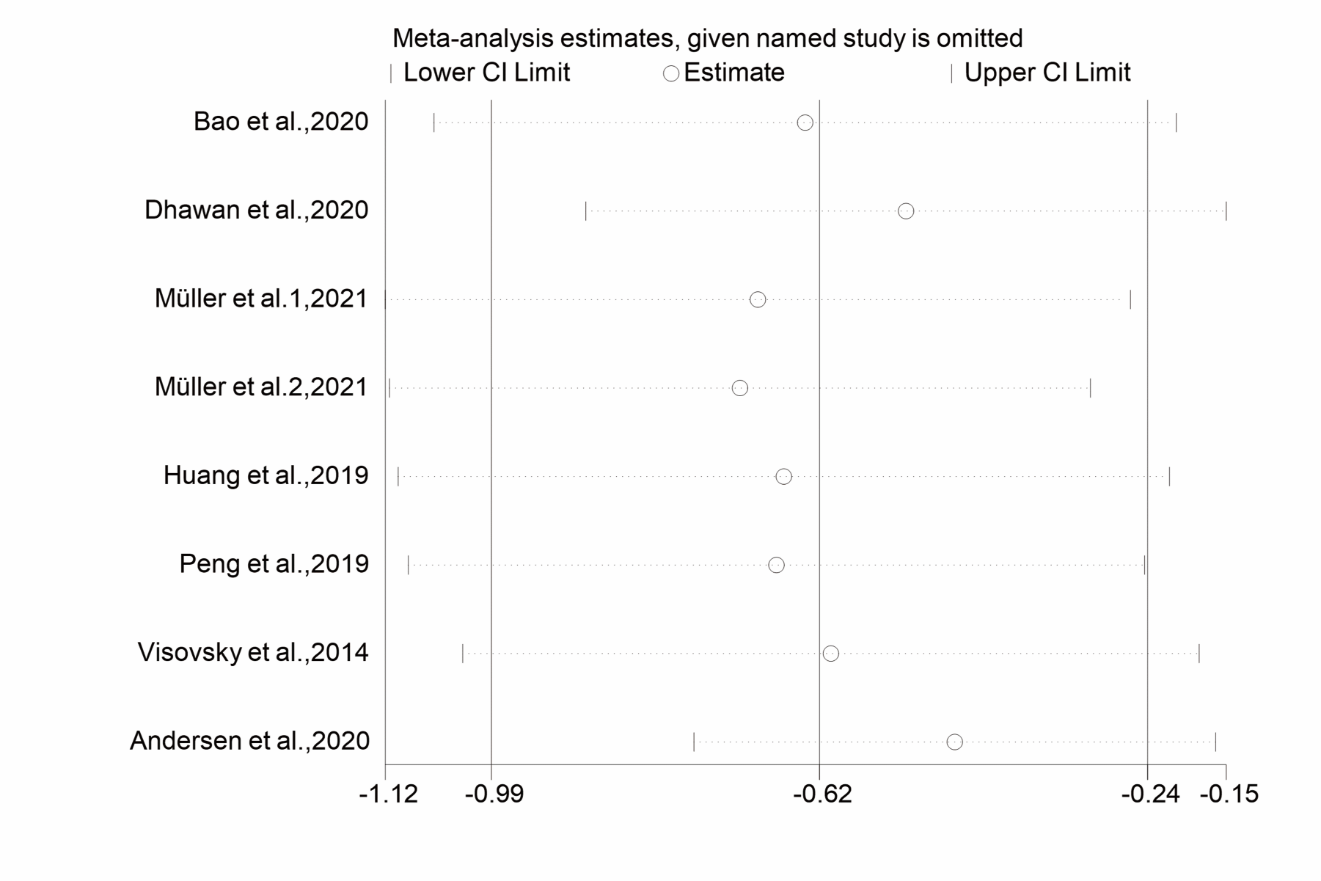


**Supplementary Figure 2.** Sensitivity analysis of the total symptom score.


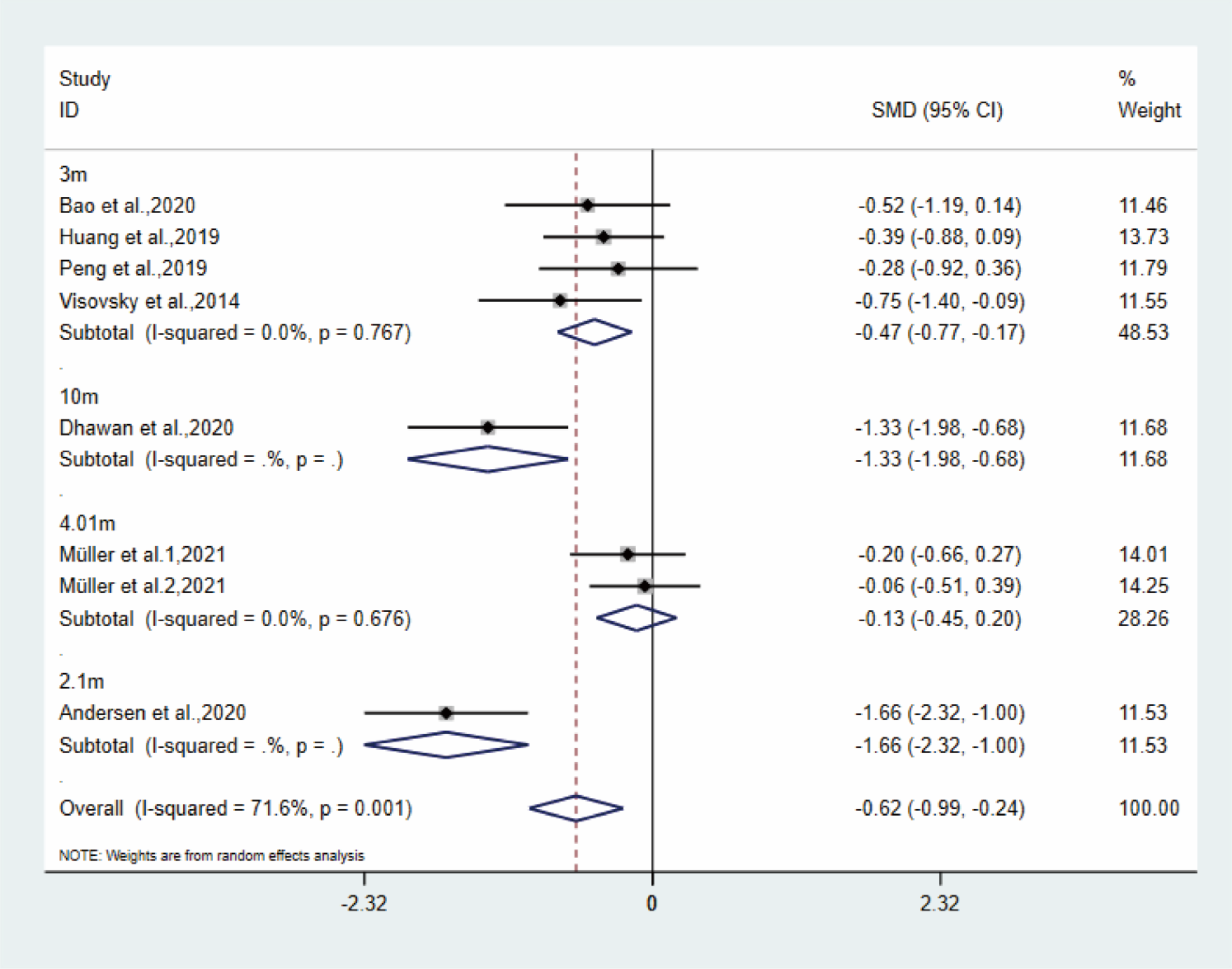


**Supplementary Figure 3.** The subgroup meta-analysis of total symptom score.
